# Orchid fruit and root movement analyzed using 2D photographs and a bioinformatics pipeline for processing sequential 3D scans

**DOI:** 10.1002/aps3.11567

**Published:** 2024-02-09

**Authors:** Dewi Pramanik, Lotta Vaskimo, K. Joost Batenburg, Alexander Kostenko, Kevin Droppert, Erik Smets, Barbara Gravendeel

**Affiliations:** ^1^ Evolutionary Ecology Naturalis Biodiversity Center Darwinweg 2 2333 CR Leiden The Netherlands; ^2^ Institute of Biology Leiden, Faculty of Science Leiden University Sylviusweg 72 2333 BE Leiden The Netherlands; ^3^ Research Center for Horticulture, Research Organization for Agriculture and Food National Research and Innovation Agency (Badan Riset dan Inovasi Nasional/BRIN) Cibinong Science Center, Jl. Raya Jakarta‐Bogor, Pakansari, Cibinong West Java 16915 Indonesia; ^4^ Faculty of Science and Technology University of Applied Sciences Leiden Zernikedreef 11 2333 CK Leiden The Netherlands; ^5^ Leiden Institute of Advanced Computer Science, Faculty of Science Leiden University, Snellius Niels Bohrweg 1 2333 CA Leiden The Netherlands; ^6^ Computational Imaging Centrum Wiskunde en Informatica Science Park 123 1090 GB Amsterdam The Netherlands; ^7^ Ecology, Evolution and Biodiversity Conservation, KU Leuven Kasteelpark Arenberg 31, BOX 2435 3001 Leuven Belgium; ^8^ Radboud Institute for Biological and Environmental Sciences Radboud University Heyendaalseweg 135 6500 GL Nijmegen The Netherlands

**Keywords:** *Erycina pusilla*, fruits, high‐precision laser scanning, *Phalaenopsis equestris*, plant growth, resupination, roots, twisting

## Abstract

**Premise:**

Most studies of the movement of orchid fruits and roots during plant development have focused on morphological observations; however, further genetic analysis is required to understand the molecular mechanisms underlying this phenomenon. A precise tool is required to observe these movements and harvest tissue at the correct position and time for transcriptomics research.

**Methods:**

We utilized three‐dimensional (3D) micro–computed tomography (CT) scans to capture the movement of fast‐growing *Erycina pusilla* roots, and built an integrated bioinformatics pipeline to process 3D images into 3D time‐lapse videos. To record the movement of slowly developing *E. pusilla* and *Phalaenopsis equestris* fruits, two‐dimensional (2D) photographs were used.

**Results:**

The *E. pusilla* roots twisted and resupinated multiple times from early development. The first period occurred in the early developmental stage (77–84 days after germination [DAG]) and the subsequent period occurred later in development (140–154 DAG). While *E. pusilla* fruits twisted 45° from 56–63 days after pollination (DAP), the fruits of *P. equestris* only began to resupinate a week before dehiscence (133 DAP) and ended a week after dehiscence (161 DAP).

**Discussion:**

Our methods revealed that each orchid root and fruit had an independent direction and degree of torsion from the initial to the final position. Our innovative approaches produced detailed spatial and temporal information on the resupination of roots and fruits during orchid development.

A change in the spatial orientation or conformation of an organ or its parts is defined as plant movement (Sisodia and Bhatla, 2018). Most plants grow linearly or circumferentially (Steeves and Sussex, 1989), but others grow in non‐linear directions, such as in twists and spirals, known as helical growth. Here, twisting is defined as a cross‐sectional rotation around a plant's midline (Otsuka and Tsukaya, 2021). Several terms, each with a slightly different denotation, have been applied to stimulus‐induced twisting, including tropism (Snow, 1962), heliotropism (Atamian et al., 2016), torsion (Borchers et al., 2018), circumnutation (Fiorello et al., 2020), and resupination (Dines and Bell, 1994; Harley et al., 2017). Plant resupination has been reported in leaves (Hill, 1939; Lyshede, 2002; Hofreiter and Lyshede, 2006), flowers (Hill, 1939), inflorescences (Hill, 1939; Niu et al., 2016), and fruits (van Wyk and Albrecht, 2008).

Resupination is viewed as a “trademark” characteristic (Arditti, 2002) of the Orchidaceae (Dressler, 1981), with most research into this process focused on orchid flowers. The term resupination originates from the Latin word “resupinus,” which means “facing up.” Resupination, a 180° twist during plant development, brings what would be the basal part of an organ to the top and the most apical part to the bottom. In unopened orchid floral buds, the labellum is often situated above the gynostemium, but as the flower opens, the labellum twists to become ventral to the gynostemium (Ames, 1938; van der Pijl and Dodson, 1966; Arditti, 1980). The resupination of orchid flowers improves pollination by providing landing platforms for pollinators, exposing the labella to sunlight for an optimal display of ultraviolet or color patterns, increasing the temperature of the labella to volatilize scents, and providing space for the opening flowers (Ernst and Arditti, 1994; Arditti, 2002; Johnson and Hobbhahn, 2010). The orientation of resupinated flowers is 180°, but in some orchid species the flowers can undergo double resupination (360°). In *Dendrobium lineale* Rolfe, young floral buds often reorient themselves during resupination (Indraloka and Purnobasuki, 2019), with the bud making a 180° turn so the labellum faces downwards (Nyman et al., 1984, 1985; Indraloka and Purnobasuki, 2019; Netlak et al., 2022). By contrast, *Angraecum eburneum* Bory and *Malaxis paludosa* flowers experience double resupination as they twist over 360° during development (Ames, 1938; Hill, 1939; Yam et al., 2009). Resupination does not always occur in the floral bud stage; the petals, medial sepal, and lip of *Bulbophyllum praetervisum* J. J. Verm. undergo resupination during blooming in response to the environment (Indraloka and Purnobasuki, 2019).

Even though many reports of resupination focus on orchid flowers, their inflorescences and fruits also resupinate (Ames, 1938), while in other plants, resupination is also reported for leaves (Hill, 1939; Chitwood et al., 2012). Resupination provides several advantages during development. Leaves resupinate to optimize sunlight uptake by rotating into angles such that both sides of the leaf area are equally exposed to sunlight. Fruits resupinate to face different directions to maximize seed dispersal. Aerial roots also twist for optimal positioning to absorb sunlight, nutrients, and water from their environment, which is why we use the term resupination for this movement.

Orchid organs can rotate in different directions and different degrees relative to a vertical plane. Here, three different terms are used to describe the various movements of orchid roots and fruits. The term “bending” is used to reflect a change in fruit orientation in the vertical plane. “Twisting” is used for turning or rotating along the horizontal axis for less than 180°, whereas “resupination” refers to turning or alternating between 180° and 360° along the horizontal axis. The direction and degree of rotation can vary from one organ to another within an individual plant or between different plants.

Most studies on orchid resupination have involved morphological observations only. Early studies depicted plant development through simple drawings of resupinating roots, leaves, flowers, and fruits. More detailed studies of resupination using time‐lapses were made possible by the invention of photography and, subsequently, video. The disadvantage of the latter two techniques is that the resupinating organ could rotate out of view, rendering the recordings only partially informative. Moreover, such methods are inadequate for more detailed examinations. The advancement of genetic research requires information about the precise moment of start and end of resupination to determine the genes responsible for initiating this process. A high‐precision tool is therefore needed for observing resupination in such detail that tissue can be harvested at the correct position and time for RNA extraction and transcriptomic analyses. Combining three‐dimensional (3D) computed tomography (CT) with transcriptomic analysis to identify these genes facilitates the elucidation of the entire picture of the development and evolution of the underlying genetic mechanisms. This combination was previously used to successfully link the ontogeny of orchid floral organs and structures to their developmental genes (Dirks‑Mulder et al., 2017; Pramanik et al., 2020). In this paper, we employed two‐dimensional (2D) photography and 3D CT to track the movement of orchid roots and fruits. The resulting information could be used to select timepoints and specific tissues to target in future genetic studies capturing genes expressed from the onset of resupination until the end via transcriptomics.

Three‐dimensional CT was originally developed to investigate human bodies, generating 3D reconstructions of internal anatomy that can aid surgeons and doctors in performing complex medical procedures. This technique uses a series of X‐ray images acquired from a range of angles to computationally reconstruct a 3D volumetric image of the X‐ray attenuation throughout the scanned object. Three‐dimensional CT can also make very accurate reconstructions of an entire plant, to the tissue level, while barely damaging the specimen, so that development can be followed through time. This technique has also been used to make images of living plant root meristems (Jerominek et al., 2014), determine floral stages (Tracy et al., 2017), and investigate the evolutionary origin of plant organs (Staedler et al., 2013; Karahara et al., 2015; Dirks‑Mulder et al., 2017, 2019; Heiduk et al., 2020; Pramanik et al., 2020), to name just a few examples. The application of 3D CT to produce time‐lapse videos for the study of plant development allows the investigation of various plant structures that possess 3D features that are challenging to discern from 2D images alone (Piovesan et al., 2021). Integrating 3D CT and time‐lapse videos offers additional benefits, such as facilitating the visualization and analysis of complex spatial patterns and structures within plant organs. This allows researchers to explore intricate 3D architectures, spatial relationships between different cell types, and the movements and interactions of cellular components during organ development; therefore, 3D time‐lapse videos are particularly well‐suited for tracking the process of resupination in fast‐growing organs, such as the aerial roots of *Erycina pusilla* (L.) N. H. Williams & M. W. Chase orchids.

Software for 3D reconstruction is mostly specific to each manufacturer of 3D CT scanners. For more advanced reconstruction computations, separate software can also be used, such as the commercially available Avizo visualization and data analysis software (Thermo Fisher Scientific, Waltham, Massachusetts, USA). In addition, there are many image reconstruction packages available that offer the additional benefit of transparent, open‐source code, such as the ASTRA Toolbox (Palenstijn et al., 2017), Livermore Tomography Tools (Champley et al., 2022), and the FleXbox toolbox (Centrum Wiskunde and Informatica [CWI] [Dutch National Research Institute for Mathematics and Computer Science], Amsterdam, the Netherlands). The FleXbox toolbox is a Python tomographic reconstruction and editing software package. It can deal with different geometrical scanning settings and multiple data sets from different snapshots in time, allowing the customization of processing algorithms and producing large amounts of data (Kostenko et al., 2020). FleXbox is divided into three modules: FlexData (https://github.com/cicwi/flexDATA), FlexTomo (https://github.com/cicwi/flexTOMO), and FlexCalc (https://github.com/cicwi/flexCALC). FlexData is used to define the geometry, FlexTomo to reconstruct the tomography, and FlexCalc to prototype the pipeline (Kostenko et al., 2020). Many programs are available to visualize and record reconstructions, such as the open‐source software platform 3D Slicer (Fedorov et al., 2012), which provides an extensible platform for automated algorithms to be customized according to different needs (Nardelli et al., 2015). This software can be used to study various organs by optimizing the settings and adjusting the orbit; however, it is important to take into consideration that the reconstruction software supports customizable processing routines, flexible scanning geometries, and large data volumes (Kostenko et al., 2020). When studying the development of a specific organ, it may be necessary to tailor the available bioinformatic pipeline accordingly. In this report, we modified the FleXbox software in combination with 3D Slicer to create a new bioinformatics pipeline to generate a 3D time‐lapse video capturing the process of resupination in fast‐growing *E. pusilla* roots. In addition, we documented the development of slower‐developing *E. pusilla* and *Phalaenopsis equestris* (Schauer) Rchb. f. fruits from one day after pollination (DAP) to fruit dehiscence using 2D photographs.

Our main goals were: (1) to build a new bioinformatics pipeline to produce high‐resolution 3D time‐lapse videos to capture resupination in weekly CT scans of roots, flowers, and fruits of *E. pusilla*, and (2) to study the resupination of *E. pusilla* and *P. equestris* fruits using weekly 2D photographs. To improve our understanding of the precise timing of resupination for future genetic studies of resupination in orchids, we aimed to answer the following questions: (1) During which developmental stages of fruits (early, middle, late) and roots does resupination occur? (2) How often do orchid fruits and roots twist until they reach their final position? (3) Is there a fixed final projection of specific parts of the roots and fruits after resupination stops?

## METHODS

### Plant materials

#### Phalaenopsis equestris

*Phalaenopsis equestris* is a well‐established orchid model. It was the first plant with Crassulacean acid metabolism (CAM) to have its genome sequenced, which was around 1.6 Gbp in size (Cai et al., 2015) and distributed over 44 chromosomes. This species has a relatively long life cycle, with the fruit maturing between 140 and 180 DAP.

*Phalaenopsis equestris* plants were grown at room temperature (22°C ± 2°C) with exposure to natural light with a light intensity of 10,000–20,000 lux. The plants were positioned near a glass window, with an angle set to maximize their reception of abundant indirect light reflected from the walls. The plants were subjected to gravitropism, and mixed fertilizers were applied to the plants once every two weeks. Five flowers of three different plants were self‐pollinated, and fruit development was recorded with weekly 2D photographs until one week after fruit dehiscence over a period of six months.

#### Tissue culture of *Erycina pusilla*

*Erycina pusilla*, an emergent orchid model, has a relatively small genome of ca. 1.475 Gbp distributed over only six chromosomes (Felix and Guerra, 1999; Chase et al., 2005). The species develops relatively quickly, with fruit development occurring on average 150 days after germination (DAG), and can be grown in vitro, making it an easy orchid to study (Dirks‑Mulder et al., 2017).

*Erycina pusilla* plants were grown in tissue culture conditions in growth chambers (model SGC120; Weiss Technik, Loughborough, United Kingdom) at the Naturalis Biodiversity Center in Leiden, the Netherlands, at a constant temperature (22°C) and humidity (50%). Each of the four shelves of the growth chambers holds three luminescence tubes (Philips Master TL‐D 36W/840 8A; Philips, Amsterdam, the Netherlands). This type of lamp emits visible light across the entire spectrum, including blue light (400–500 nm), green light (500–600 nm), and red light (600–700 nm). The plants were exposed to a consistent 12‐h light regimen to induce gravitropism. The light intensities measured between the lamp and the culture tubes at distances of 8, 10, 14, and 20 cm were 6280, 6250, 5230, and 4780 lux, respectively.

The *E. pusilla* tissue culture cycle consists of four steps: (1) preparation of culture media, (2) self‐pollination to produce fruits, (3) sterilizing the fruits and sowing seeds in a multiplication medium, and (4) multiplication and maintenance of plantlets. As the plantlets mature and flower, the cycle restarts with the first step. The entire cycle spans approximately six months. Each step is described in more detail below.

Two media types were used: a multiplication medium and a maintenance medium. The multiplication medium was prepared by mixing Phytamax Orchid Multiplication Medium (P6793; MilliporeSigma, Burlington, Massachusetts, USA) with 20 g/L banana powder (B1304.005; Duchefa Biochemie, Haarlem, the Netherlands) and 2 g/L charcoal (C1302; Duchefa Biochemie) (pH adjusted to 5.7–5.8). Before autoclaving, 4 g/L Gelrite (G1101.0500; Duchefa Biochemie) was added to solidify the medium as a final step. The medium was sterilized in a 1000‐mL Duran bottle (Duran laboratory bottles with caps [item #Z305200]; Merck, Darmstadt, Germany) by autoclaving the individual components at 121°C with 10.35 pascals for 20–25 min. Sterile media was then poured into the plastic culture container (10 cm base diameter × 8 cm tall) (O118/80 + OD118 with XL filter; Eco2box, Hasseltkouter, Belgium). Each container contained approximately 20 mL of media.

The maintenance medium (Duchefa Orchimax medium [O027]; Duchefa Biochemie) was prepared by adding banana powder, charcoal, and Gelrite in the same amounts described above. The medium was sterilized by autoclaving the individual components at 121°C with 10.35 pascals for 20–25 min. Following autoclaving, the media was poured into individual culture tubes (item #W1607; Duchefa Biochemie). Before being used for culture, all media was stored at room temperature for 3–7 days to assess fungal or bacterial contamination in the media.

To obtain *E. pusilla* fruits through self‐pollination, the mature fruits were harvested approximately 98–112 DAP. They were then sterilized in 0.5% sodium dichloroisocyanurate (NaDCC [item #218928]; Merck) for 40 min, followed by a 1‐min wash in 0.01% NaDCC. Seed capsules were opened in a liquid multiplication medium containing 0.03% NaDCC and 2 mL/L plant preservative mixture (P820; Apollo Scientific, Stockport, United Kingdom). Subsequently, 1–1.5 mL of the seed solution was transferred to a solid culture multiplication medium for germination within 28–60 days. After germination, seedlings were subcultured on the same multiplication medium. For *E. pusilla*, we define a young plant as between 7 and 84 DAG; plants at this stage typically have 1–2 leaves and 1–2 small roots. We define the mature stage as 91 DAG; these plants have a developed root system and flower spike.

Mature plants were moved to individual culture tubes on a maintenance medium. Once mature plants produced flowers, a total of three flowers from three different plants were self‐pollinated, and fruit development was followed with weekly 2D photographs over 5–6 months until one week after fruit dehiscence (161 DAP).

#### *Erycina pusilla* samples used

A total of 19 *E. pusilla* plants were divided into three groups for medium testing (*N* = 6), actual scanning (*N* = 9), and a control (*N* = 4) not exposed to any X‐rays. For the medium testing, plants harvested at 140 DAG were placed in different media to discover the optimal combination of nutrition and materials for achieving a good contrast and providing the plant with sufficient nutrients to survive long‐term scanning. The media tests consisted of Gelrite, glass beads, sterilized sand, solid agar, cotton, and *Sphagnum* moss, of which the latter was found to be most optimal (Figure S1 in Appendix S1; see Supporting Information with this article). Of the plants used for scanning, one was a young plant, and the remaining four were mature (>84 DAG). These samples are named according to their developmental stage, and the tested features are described below (under “Scanning of live plants”).

### Scanning of live plants

Individual plants were placed into red‐capped Falcon containers (180‐mL Polypropylene Straight Container with screw cap [item #15428844]; Gosselin, Borre, France) and situated in a holder in the chamber of the CT scanner to prevent distortion during scanning.

A Zeiss Xradia (520 Versa 3D X‐ray microscope; Carl Zeiss, Oberkochen, Germany) and MicroCT SkyScan (SkyScan 1172; Bruker, Billerica, Massachusetts, USA) were used to scan the *E. pusilla* plants. The software accompanying the Xradia was the Zeiss Xradia Versa Scout‐and‐Scan Control System (version 14.0.14829.38124; Carl Zeiss). The software used for the SkyScan was SkyScan 1172 version 1.5.26 (Bruker). Several actions were taken prior to scanning. When the X‐ray generator of the SkyScan was turned off for more than 8 h, the X‐ray generator had to warm up for approximately 15 min. After this, a protocol was entered by adjusting the kilovolts to the desired level. To scan the *E. pusilla* plants, a voltage ranging between 30 and 40 kV was used depending on the size of the plant scanned, combined with 175–250 mA power. The camera size was set to medium (2000 × 1336 pixels). Prior to each scan, a Flat‐Field reference was made, with the correct settings corresponding to those used to scan the plant in question (see Table S1 in Appendix S1). Each plant was scanned once a week, with a total scanning duration of approximately 1 h for young plants and 2 h for mature plants. All mature plants were scanned at the same time of day so that the recorded movement reflected exposure to consistent light conditions. A detailed step‐by‐step scanning protocol can be found in Appendix S2.

After scanning, the individual images were combined into a 3D model using NRecon software (Carl Zeiss), which builds reconstructions of all the raw CT scans. For each section, a post‐alignment step and fine‐tuning were performed. The software aligns the images during this fine‐tuning step in multiple ways, and the best of these alignments was chosen for each section (Appendix S2).

### 3D time‐lapse pipeline

**Figure 1:**
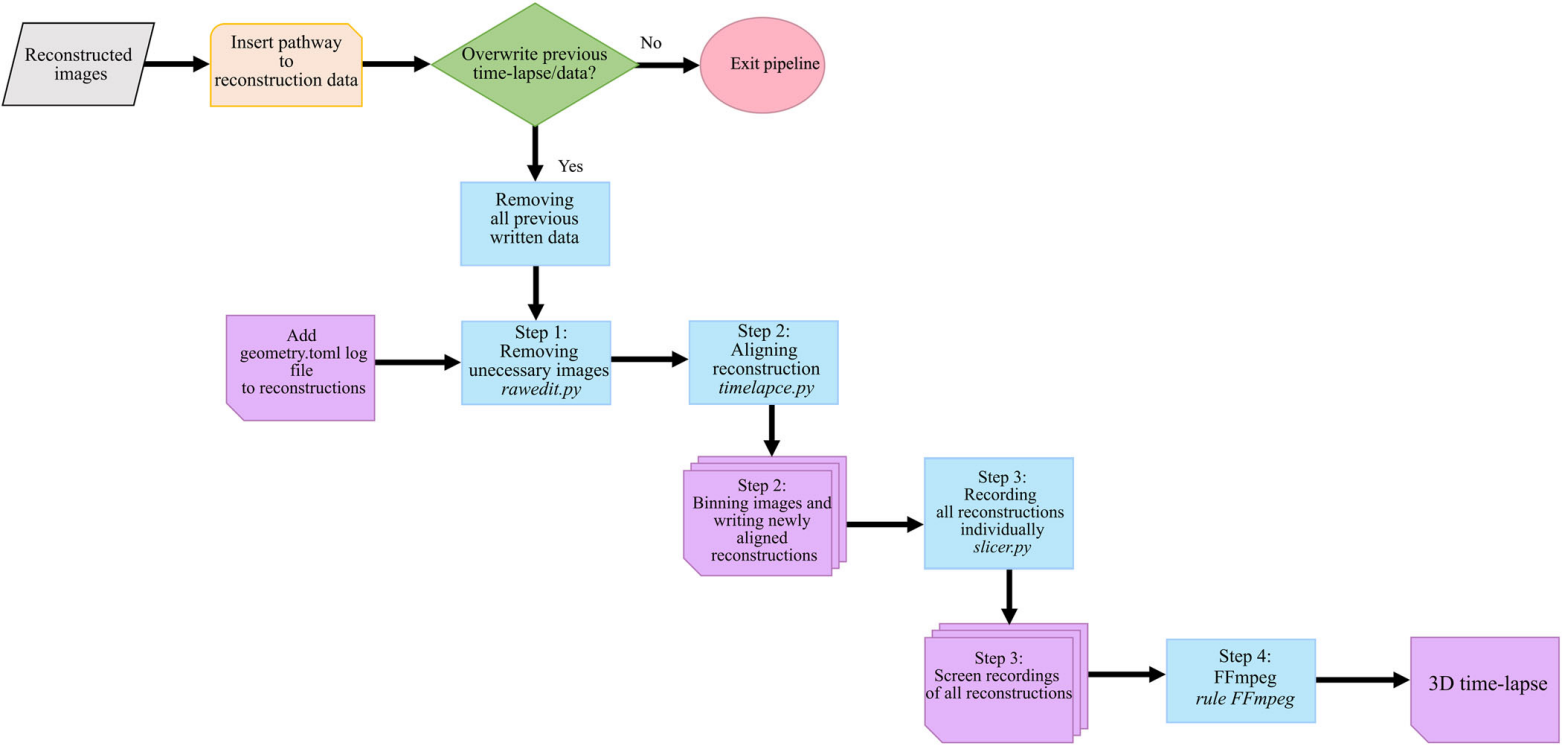
Detailed flowchart of our 3D time‐lapse pipeline. To run the pipeline, first open the shell and go to the correct directory (3D Lapse Pipeline folder). The full pipeline code command can be run by entering the Snakemake command. After starting the pipeline, the pathway to the folder containing all reconstructions must be manually entered in order to continue (orange box). The pipeline checks whether the folder of recontruction data has been run before (green box); if it has, a decision is made whether to continue (yes) or abort (no). If the decision is yes, the pipeline moves on to Step 1, in which the raw edit script (*rawedit.py*) processes the data, trims the images to equal volumes, and incorporates *geometry.toml* to generate one or multiple log reconstruction data sets. Step 2 involves using the time‐lapse script (*timelapce.py*) to align image reconstructions, bin images, and produce binned images and new reconstructions. In Step 3, the 3D Slicer script (*slicer.py*) is employed to visualize image reconstructions and record rotating reconstructions, resulting in screen recordings of all reconstructed data. Finally, Step 4 uses the FFmpeg command to compile the screen recordings into an image sequence or time‐lapse video. The output is a 3D time‐lapse video. Color codes: gray = data; orange = manual input; green = decision; blue = process; purple = one or multiple documents generated; red = end of pipeline.

A pipeline was built using in‐house software provided by the CWI (Figure 1). This pipeline was designed to run on Linux systems. We used Python 3.7 (Python Software Foundation, Wilmington, Delaware, USA) as the programming language, as this version was compatible with FleXbox (Kostenko et al., 2020). Several scripts were supplied within the FlexCalc module to explain how the package works. One of the six example scripts (*ex6_timelapce.py*) was used and modified further. The master pipeline is available here: https://github.com/LMVaskimo/3D-Lapse-Pipeline/tree/master/3D%20Lapse%20Pipeline (see Data Availability Statement), while detailed information about the pipeline can be found in the README.md file (https://github.com/LMVaskimo/3D-Lapse-Pipeline/blob/master/README.md).

#### Working with 3D Slicer

Before using the 3D Slicer script, we used the visualization software Avizo version 9.2 to manually create 3D time‐lapse videos (Videos 1 and 2) of the reconstruction data set. However, as Avizo software is not open access, we decided to abandon Avizo and use freely available 3D Slicer version 4.10.2 to automate 3D time‐lapse making.

**Video 1:** The 3D time‐lapse video of a young *E. pusilla* plant, produced using Avizo.

**Video 2:** The 3D time‐lapse video of a mature plant, produced using Avizo.

The preparation step in the pipeline was to convert all scripts into lines of code in a Python script using the built‐in Python application programming interface (API), resulting in a new Python script to visualize and record 3D time‐lapses. First, using 3D Slicer, the reconstructed data (Data 1 and 2, see Data Availability Statement) were opened as volumes, which were converted into 3D models. All the reconstructions were loaded into 3D Slicer using a Python script and placed in sequence to create a 3D time‐lapse animation. The contents of the time‐lapse were made visible using volume rendering. Screenshots were made of every reconstruction using the built‐in Screen Record module. These were later merged into the desired 3D time‐lapse video using FFmpeg (FFmpeg Developers, 2016). A step‐by‐step operation guide for this pipeline is presented in the Naturalis pipeline help file (https://github.com/LMVaskimo/3D-Lapse-Pipeline/blob/master/3D%20Lapse%20Pipeline/naturalis_pipeline_help.txt).

#### Running the pipeline

Snakemake (version 5.10; Mölder et al., 2021) was used to stitch all separate scripts together. Piping software was used to develop the backbone of the pipeline. This pipeline facilitated the use of the scripts, as only the SnakeFile needed to be run to program all the below‐mentioned scripts in a sequence. The flowchart depicted in Figure 1 is a detailed overview of the SnakeFile when run. The SnakeFile consists of five rules: *rule all*, *rule raw_edit*, *rule timelapse*, *rule slicer*, and *rule help*. First, users should open the shell and go to the correct directory (3D Lapse Pipeline folder) to run the pipeline. The full pipeline code command can be run by entering: ‘snakemake ‐s Minisnake ‐‐use‐conda ‐T ‐R al’ or by using: ‘snakemake ‐s Minisnake ‐‐use‐conda ‐T <insert rule > ’ to run a single rule. Only the given rule and everything dependent on it will run. After starting the pipeline, the pathway to the folder containing all reconstructions must be entered in order to continue. The pipeline will detect whether it has been run before. If this is the case, a decision will be made to continue or abort. When the choice to continue is selected by typing “yes,” it will remove all previously written data originating from the previous run, including the previously generated time‐lapse. It is advised to copy the previously generated time‐lapse before this point. After this, the pipeline will run all rules without any interruptions.

These rules correspond to their similarly named scripts. All rules, excluding *rule all*, are displayed in Figure 1, with each step referring to a rule and a script in the SnakeFile. The rules are discussed in more detail below.

#### Rule all

This rule was created to ensure that all the rules in the pipeline would run by having every output file functioning as an input file for the next step. Here, the wildcards of the data are defined. These wildcards consist of all reconstructions made. This rule is not displayed on the flowchart, as it has no additional functionalities other than aiding the running of the rules in the proper order.

##### Step 1: Rule rawedit *and the* rawedit.py *script*

Once the pathway is entered, the first pipeline rule, *rawedit* (called *rawedit.py*), is activated to run a Python script (https://github.com/LMVaskimo/3D-Lapse-Pipeline/blob/master/3D%20Lapse%20Pipeline/rawedit.py). The script was developed for data pre‐processing to ensure that the data generated are compatible with the script in the next step. Adjusting data compatibility was necessary due to using the micro‐CT scanner, which did not have a high enough X‐ray detector range to fit the entire sample in the field of view. An oversized scan had to be used to fit the entire sample in the field of view, which caused the reconstructions to be different sizes. To bypass this issue, the *rawedit.py* script was written. The *rawedit.py* script skimmed through all the reconstruction folders and checked whether all the reconstructions had the same dimensions, as this was necessary for the next *timelapce.py* script to work. If this was not the case, all reconstructions were trimmed from the top by removing images until they all had the same volume, and the *geometry.toml* was added to the folder if an image was missing (https://github.com/LMVaskimo/3D-Lapse-Pipeline/blob/master/3D%20Lapse%20Pipeline/Geometry/geometry.toml). This step was run separately for every reconstruction using wildcards, indicated in *rule all*. FlexData was used to alter the positioning of the reconstruction to fit the predecessor's volume for the alignment of reconstructions. FlexCalc was mainly used because of its valuable capabilities for time‐lapse development. FlexCalc places all reconstructions in a sequence and ensures that adaptations are applied to all reconstructions.

##### Step 2: Rule timelapse *and the* timelapce.py *script*

After pre‐processing the images, the second rule (*rule timelapse*), containing Python script *timelapce.py*, was run (https://github.com/LMVaskimo/3D-Lapse-Pipeline/blob/master/3D%20Lapse%20Pipeline/timelapce.py). This script used the FleXbox software package to register the volumes and align the images in similar positions. After this, new slices from bottom to top were made by binning eight times. Binning refers to grouping neighboring pixels or voxels in an image to create larger pixels or voxels. It has several functions, such as reducing the overall size of the image data, reducing the effect of noise in the image data, speeding up processing, and enhancing the visual representation of the reconstructed images. This script is part of the FlexCalc module (https://github.com/LMVaskimo/3D-Lapse-Pipeline/tree/master/3D%20Lapse%20Pipeline/flexcalc).

##### Step 3: Rule Slicer *and the* slicer.py *script*

The third rule, named *rule Slicer*, was applied using the Python script *slicer.py*, and the code to run 3D Slicer was: ‘./Slicer‐4.10.2‐linux‐amd64/Slicer.exe ‐‐python‐script 3dslicer.py’. This script was run to build the 3D time‐lapse and was developed using the 3D Slicer Python API, which provides unique functionalities specifically for visualizing and recording 3D volumes. A command remotely opens the 3D Slicer visualization software to run the Python script, which displays and records all reconstructions one by one using the following command: ‘Slicer –python‐script slicer.py’. The reconstructions are first visualized using Volume Rendering. The built‐in module Screen Record is used to make screenshots of all reconstructions while rotating them after each screenshot.

##### Step 4: Rule FFmpeg

The final rule of the pipeline, called *rule FFmpeg*, used an FFmpeg command. FFmpeg places all previously made screenshots in a sequence to create a video in .mp4 format. The script of *rule FFmpeg* was: ‘ffmpeg ‐i./SlicerCapture/Screenshots/capture_%d.png ‐r 1/5./Movies/3D_lapse.mp4’, which was used as a shell command to run FFmpeg on the made screenshots and convert them into a 3D time‐lapse video.

#### Output: 3D time‐lapse video

The time‐lapse was constructed by combining a Python script run in the shell to remotely master the making of recordings of all reconstructions in 3D Slicer and an FFmpeg shell command that converted all recordings into a 3D time‐lapse (Figure 1). These time‐lapses were examined in greater detail. When an organ starts to resupinate, the exact weeks of torsion could be noted. Snapshots were created where resupination took place. The time‐lapse included the scan numbers to make it easier to keep track of the exact time of movements and the start and end of individual movements. Additionally, an automatically generated digital log file was created to facilitate the tracking of significant developmental changes.

### *Phalaenopsis equestris* fruit development

The fruit development of *P. equestris* was studied for comparison with fruit movement. Anatomical observations were conducted on different fruit stages of *P. equestris* at 0, 1, 2, 4, 8, 16, 32, 64, 120, and 186 DAP. Fruits were harvested from seven different plants cultivated in the climate room of the Naturalis Biodiversity Center. Three biological replicates were prepared for each development stage. Freshly harvested fruits were immediately immersed in 70% ethanol and stored for at least a week. For the histological analysis, anatomical fruit slices were prepared using the London Resin white embedding technique, following the method described by Pramanik et al. (2022). Images of each slice were captured using an automatic stacking microscope (Carl Zeiss). The number of cells and valve areas were counted and measured in fruit of different development stages (samples from 0–64 DAP) (Tables S3 and S4 in Appendix S1). Manual cell counting was performed by counting each cell on a fruit valve printed on A3 paper. The area of the valve was measured using ImageJ version 1.53c (Schneider et al., 2012). The counting and measurements were conducted for three biological replicates (three different fruits) and three technical replicates (three slices per individual fruit) (see Tables S3 and S4 in Appendix S1). The mean and standard error of cell numbers were calculated, and a graph was made in Excel version 16.74 (Microsoft, Redmond, Washington, USA). The main morphological changes observed during *P. equestris* fruit development included fruit elongation, cell division and growth in the fertile and sterile valves, an increase in volume, the development of six pollen tube bundles, the formation of dehiscence zones, and seed development (see Figure S5 in Appendix S1).

### Two‐dimensional photographs of *P. equestris* and *E. pusilla*

The fruit development of *P. equestris* and *E. pusilla* was recorded with 2D photographs made with a Nikon (Tokyo, Japan) D300S camera with an AF‐S Micro Nikkor 40‐mm f/2.8 G DX lens (Nikon). For *P. equestris*, we observed 15 pollinated flowers from three different plants from 1 DAP to a week after fruit dehiscence (161 DAP). For *E. pusilla*, we followed the development of three pollinated flowers from three different plants from 7 to 63 DAP. Photos were taken each week, from 7 DAP to a week after dehiscence. To ensure the consistent capture of each fruit with the same position and picture quality, we used a copy stand (Kaiser Copy Stand RSX; Kaiser Fototechnik, Buchen, Germany) equipped with a camera arm (Kaiser RPT; Kaiser Fototechnik) and lighting (Kaiser LED Lighting Unit RB 5020 DS2 5467; Kaiser Fototechnik). For each fruit sample, the camera was positioned on the camera arm at a specific angle and position relative to the column of the copy stand. A fixed point on each developing fruit sample was also marked for reference. The focal length was set to 40.00 mm (equivalent to 60 mm in the 35‐mm format), the exposure was set to 0.5 s, the aperture was adjusted between f/13 and f/22, and the ISO was set to 400. Detailed image‐capture settings were noted for each fruit sample. Fruit development was recorded until dehiscence.

Throughout this period, we meticulously documented the development and orientation of the fruits. We categorized fruit development into three distinct stages: early, middle, and mature development. During the early stage, the fertilized ovary undergoes changes such as cell division and differentiation, the development of six pollen tube bundles, and the formation of dehiscence zones. In the middle stage, the fruit continues to grow and undergoes further changes in size and shape, as well as the shrinkage of pollen tube bundles, cell division and growth in the fertile and sterile valves, the maturation of dehiscence zones, and trichome development. The late stage is marked by the final maturation and ripening of the fruit, including the disappearance of the pollen tube bundles, fruit shrinkage, seed maturation, and fruit dehiscence. The mature fruits have reached a maximum size and weight, changed color, and developed a softer texture. For *E. pusilla*, early fruit development occurs from 1 to 14 DAP, middle fruit development occurs from 15 to 41 DAP, and late fruit development occurs from 42 DAP until fruit dehiscence at 84 DAP (Dirks‐Mulder et al., 2019). For *P. equestris*, early fruit development occurs from 1 to 32 DAP, middle fruit development occurs from 33 to 64 DAP, and late fruit development occurs from 65 DAP until fruit dehiscence at 154 DAP (Table S5 in Appendix S1).

Fruit orientation was specifically assessed in terms of changes in the vertical plane (bending), rotation along the horizontal axis (twisting) by less than 180°, and turning or alternating around 180–360° (resupination). To quantify these changes, a protractor was used.

## RESULTS

It should be noted that the observations reported below were consistent for all specimens. The movements described for sample individuals are representive of similar results for each group.

### *Erycina pusilla* root development and resupination

Of the scanned mature *E. pusilla* plants, the majority did not produce flowers or new leaves. Instead, these plants only continued to produce new roots until they wilted. The absence of new leaves and observed wilting are likely linked to X‐ray‐induced stress. In the control group, two of the four control plants produced both new roots and flowers. The development of the scanned plants slowed down after seven weeks of scanning (Table S2 and Figure S2 in Appendix S1).

**Figure 2:**
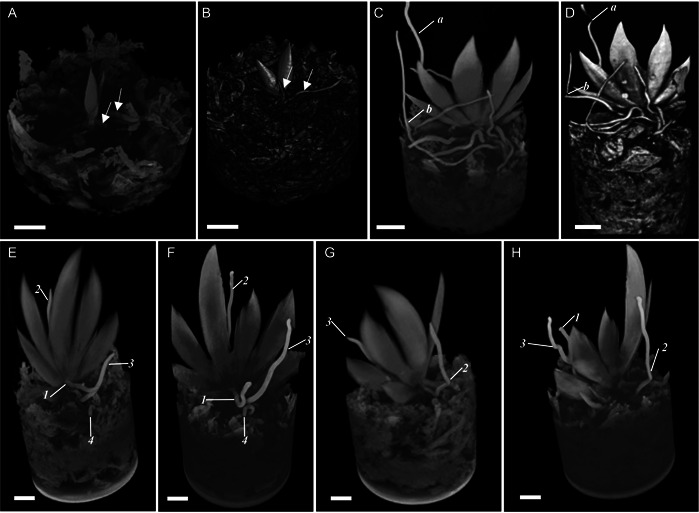
Screen recordings of two sides of a Falcon container containing a seedling (A, B), flowering plant (C, D), and non‐flowering plant (E–H) of *Erycina pusilla* during repeated micro–computed tomography (CT) scanning over a 12‐week period. (A, B) Scans of seedling roots (indicated with white arrows) that resupinated within a four‐day period, shown before (A) and after (B) resupination. (C, D) Scans of the roots of a flowering plant (indicated with “a” and “b”) that resupinated over a 12‐week period, shown before (C) and after (D) resupination. (E–H) Scans of the roots of a non‐flowering plant (indicated with 1, 2, 3, and 4) that resupinated over a 12‐week period. Panels E and F display scans of the front of a non‐flowering plant showing the roots before (E) and after (F) resupination; panels G and H show scans of the back of a non‐flowering plant before (G) and after (H) resupination. Scale bar = 1 cm.

We scanned 70‐DAG seedlings of *E. pusilla* with two leaves and three roots (Videos 1 and 3). One new leaf and several roots emerged during the first eight weeks. Prior to this, one root twisted slightly before the scanning process started (Figure 2A). At 77 DAG, a full rotation of 180° had not yet occurred in this seedling (Figure 2B). Resupination was detected in this root at 84 DAG. The seedling did not show any symptoms of wilting or death during the nine‐week scanning process, although its development was slower than the control plants (those not exposed to the X‐rays of the 3D CT), which had developed an inflorescence and flower bud by the end of this time‐lapse.

**Video 3:** The 3D time‐lapse video of a young *E. pusilla* plant, produced from a bioinformatics pipeline.

One of the three non‐flowering plants scanned had a robust root system (Videos 2 and 4). Roots of this plant had resupinated numerous times before scanning (Figure 2C). During the 13 weeks of scanning, a new leaf and resupinated roots were produced (Figure 2D); however, no additional plant growth occurred after this point, and the plant began to wilt after 15 weeks of scanning.

**Video 4:** The 3D time‐lapse video of a mature *E. pusilla* plant, produced from a bioinformatics pipeline.

In the first week of scanning a mature non‐flowering plant (Figure 2E–H), the lower roots twisted, and a leaf bud developed in the second week. Several new leaves emerged and further developed during the next three weeks of scanning. In addition, a bud appeared on the side of a leaf, indicating an emerging inflorescence. After seven weeks of scanning, the mature plant showed signs of wilting, as several leaves turned yellow then brown a week later; however, a fresh leaf had also emerged, indicating that the plant was not yet completely dead. Roots 2, 3, and 4 of the mature plant were twisted before scanning (Figure 2E, G). A new twisting cycle was observed for roots 1, 2, and 3 as the root tips started to grow in different directions. Roots 1 and 3 resupinated in one week. These roots began another cycle of resupination in week 2, which was completed in week 3. The third cycle of resupination started in week 4, where root 1 rotated in the opposing direction, whereas root 3 continued rotating in the same direction, finishing its third rotation in week 7. Root 1 had not completed its third cycle of resupination by the 12th week of scanning (Figure 2F, H).

### *Erycina pusilla* flower development and resupination

**Figure 3:**
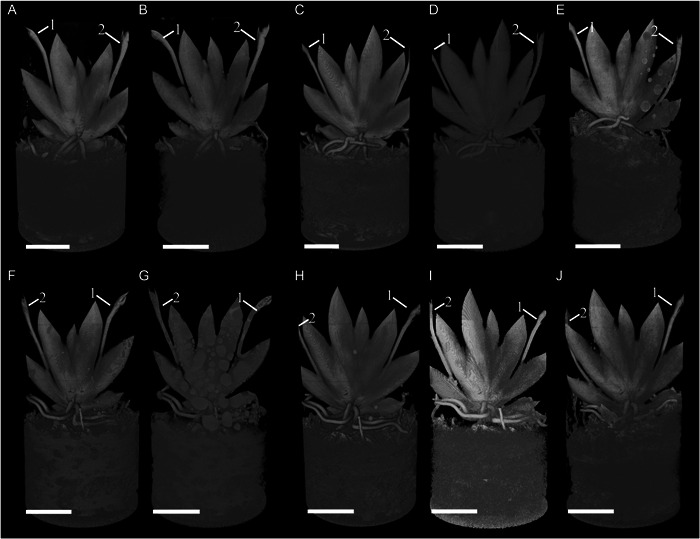
Screen recordings of two sides of a Falcon container containing a flowering individual of *Erycina pusilla*, which was repeatedly scanned using micro‐CT during a 19‐week period. (A–E) Front side after the first week (A), second week (B), fifth week (C), 12th week (D), and 19th week (E) of scanning. (F–J) Back side after the first week (F), second week (G), fifth week (H), 12th week (I), and 19th week (J) of scanning. Different inflorescence stalks are labeled with numbers 1 and 2. Scale bar = 1 cm.

In the second batch of flowering plants, we also observed the twisting of floral buds (Figure 3). In one plant, two flowers twisted 45° counterclockwise in the second week (Figure 3B, G), and had rotated 90° clockwise during the fifth week of scanning (Figure 3C, H). In a third twisting cycle, the floral buds rotated 45° clockwise during the 12th week of scanning (Figure 3D, I), resulting in a similar position as in the initial week of scanning. The final rotation cycle resulted in a 90° twist clockwise in the 19th week of scanning (Figure 3E, J).

### *Erycina pusilla* fruit development and resupination

**Figure 4:**
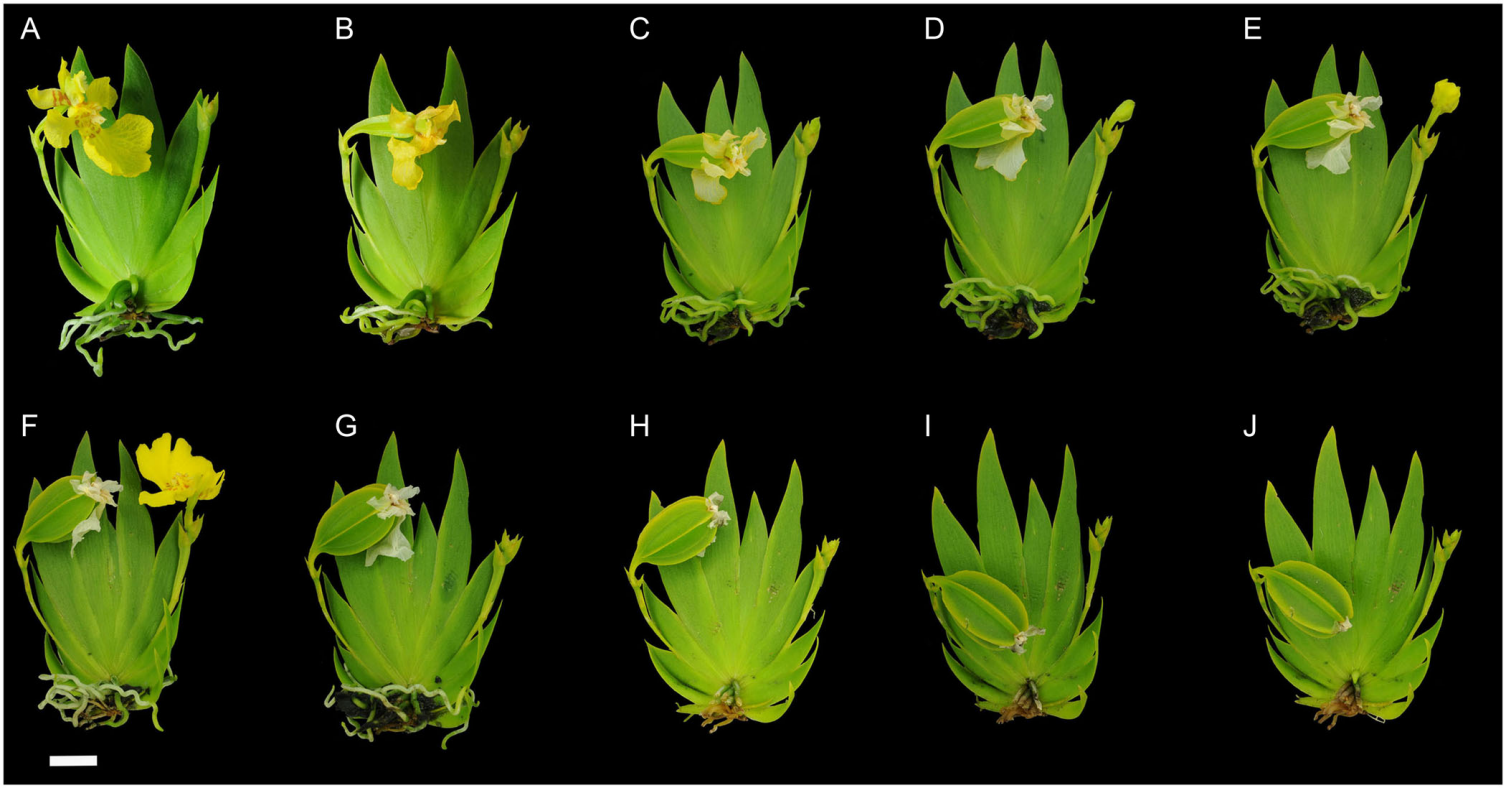
Development of *Erycina pusilla* fruit recorded in the tissue cultivation laboratory of the Naturalis Biodiversity Center, Leiden, the Netherlands. (A) Position of the flower seven days after pollination (DAP) at 67.5° from the *y*‐axis. (B, C) Developing fruit at 14 (B) and 21 (C) DAP, which bent from 67.5° to 45°. (D) Fruit bent back (counterclockwise) to a position of 67.5° at 28 DAP. (E–H) No movement was detected in the fruit at 35 (E), 42 (F), 49 (G), and 56 (H) DAP. (I, J) At 63 (I) and 70 (J) DAP, the fruit was bent clockwise to a position 270° from the *x*‐axis, and had also twisted 45°. Scale bar = 1 cm.

We observed seedling and fruit development in *E. pusilla* using 2D photographs (Figure 4, Figure S3 in Appendix S1). The fruit of *E. pusilla* both bent and twisted during development (Figure 4). In one plant, the fruit started to bend around 22.5° clockwise from 7 to 28 DAP (Figure 4A–D), after which no movement was detected from 35 to 56 DAP (Figure 4E–H). The fruit twisted a further 45° from 56 to 63 DAP (Figure 4I, J).

### *Phalaenopsis equestris* fruit development and resupination

**Figure 5:**
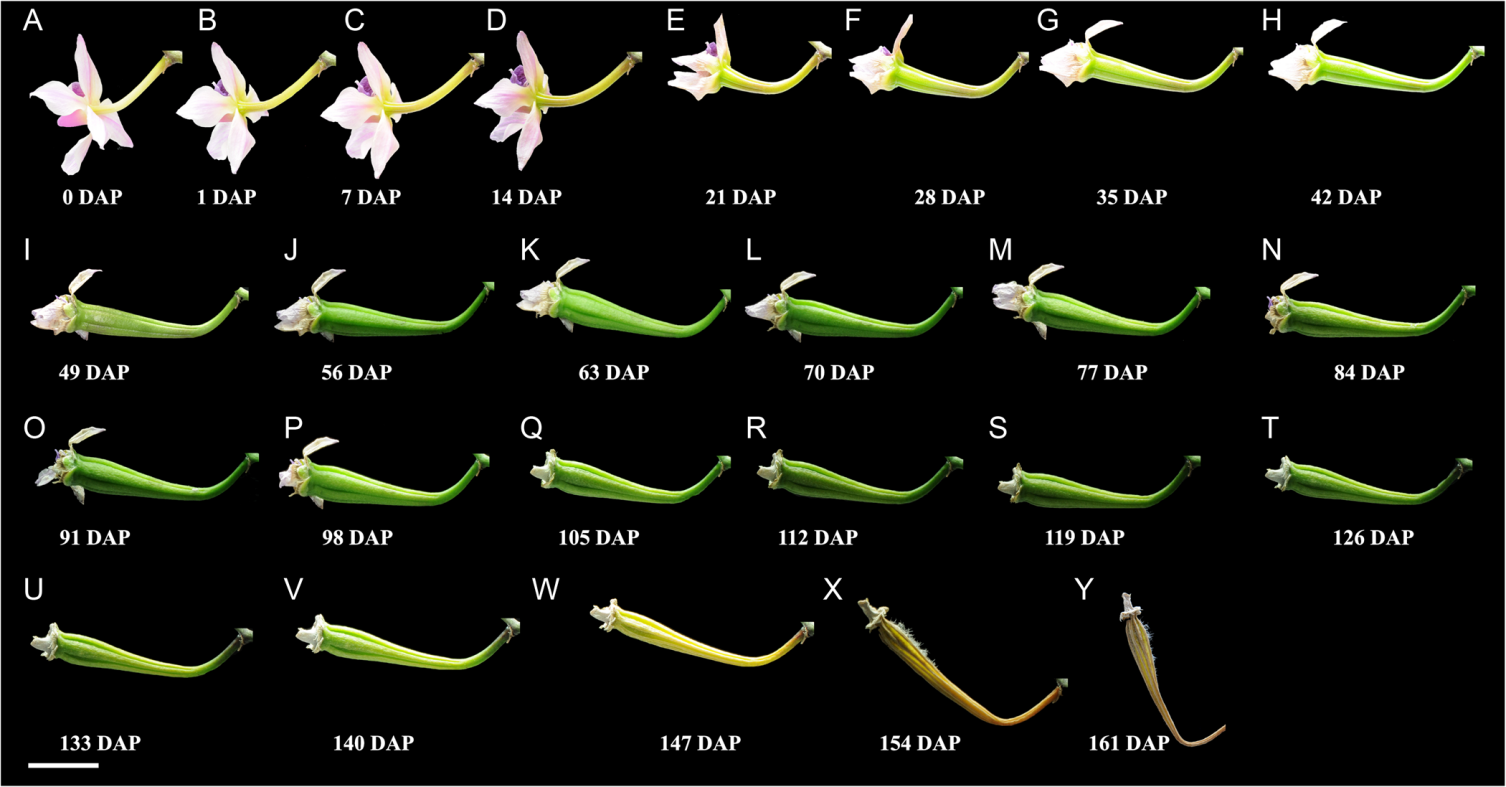
Time‐lapse images of developing *Phalaenopsis equestris* fruit in the climate room of the Naturalis Biodiversity Center, Leiden, the Netherlands. (A) The position of the pollinated flower began at 200° from the *x*‐axis. (B) The first change of fruit orientation (to 220°) was recorded at 1 DAP. (C, D) Fruit continued to bend to 180° at 7–14 DAP. (E–G) Fruit continued to bend at 21–35 DAP. (H–V) Fruit continued to bend to 155° from 42 to 140 DAP. (W) One week prior to dehiscence, the fruit twisted counterclockwise over 20°. (X) After dehiscence, the fruit twisted to 135° as compared with the *x*‐axis and 45° as compared with the *y*‐axis. (Y) The position of the fruit at 161 DAP was 110° as compared with the *y*‐axis. Scale bar = 1 cm.

Using 2D photographs, we followed the movement of *P. equestris* fruits from 1 DAP to a week after dehiscence (161 DAP) in three plants. Fruit bending occurred as early as 1 DAP. The fruits actively bent from 7 to 35 DAP and two to three weeks before fruit dehiscence (133 to 140 DAP), with orientations varying from 15° to 90° along the horizontal axis. The *P. equestris* fruits bent both clockwise and counterclockwise during development, with some bending several times but returning to their starting position before dehiscence (Figure 5).

**Figure 6:**
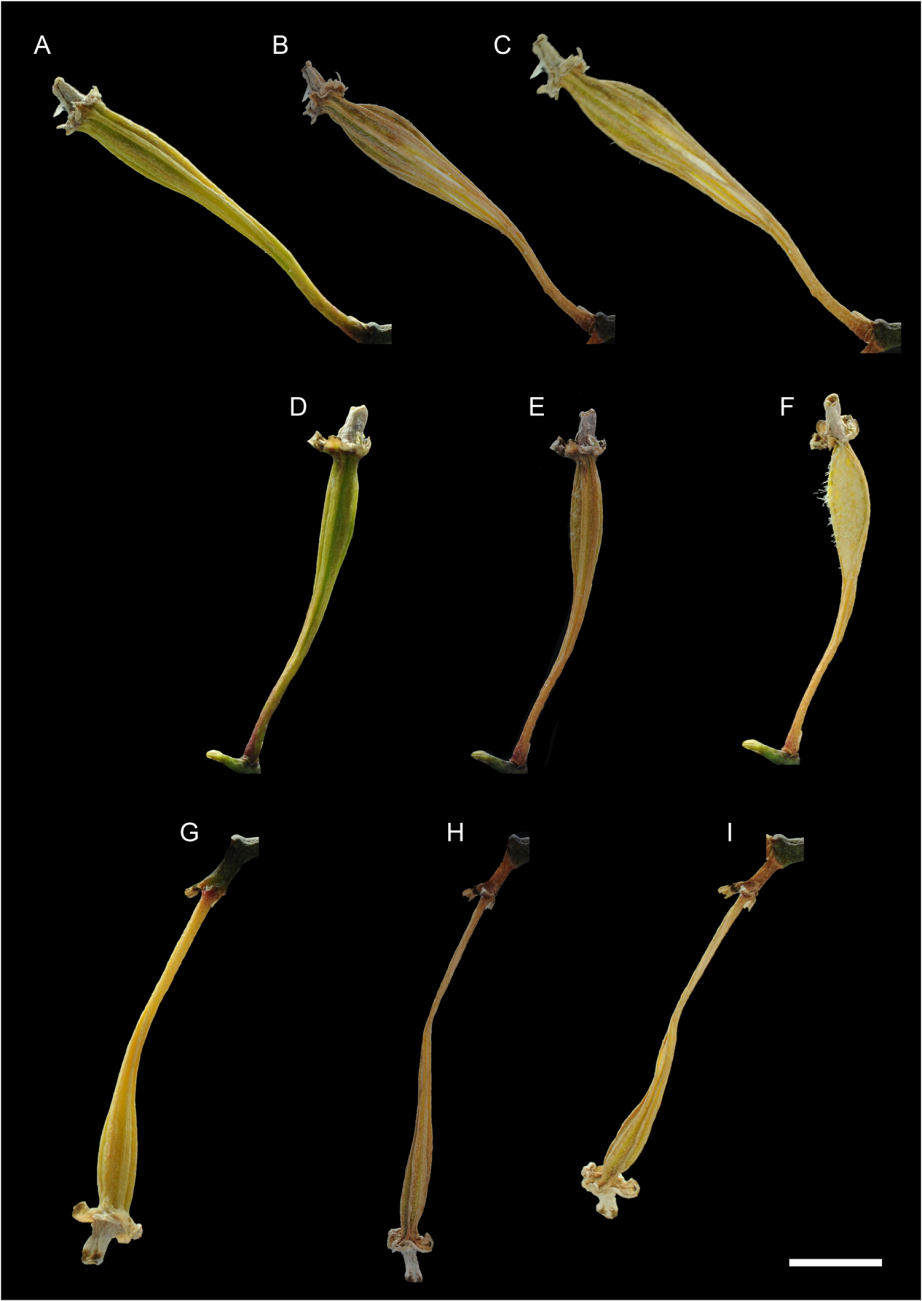
Two‐dimensional photographs recording the twisting and resupination of three different fruits of *Phalaenopsis equestris* during development. (A–C) The first fruit twisted 20° between 203 and 217 DAP. (D–F) The second fruit twisted 45° between 140 and 154 DAP. (G–I) The last fruit resupinated 180° between 203 and 217 DAP. Scale bar = 1 cm.

We also photographed *P. equestris* fruits twisting and resupinating one week before and after dehiscence. The fruits twisted 20° (Figure 5A–C), 45° (Figure 5D–F), 90° (Figure 5G–I), or 180° (Figure 5X, Y). During dehiscence, most upright and arching‐oriented fruits had their fertile valve derived from the labellum in the lower position (Figure 6A–F). For the hanging fruits, the fertile valve was at the top and the sterile valve derived from the dorsal sepal was at the bottom (Figure 6G–I). Fruit opening always occurred in one of the dehiscence zones adjacent to the fertile valve derived from the labellum (Figures 5 and 6).

## DISCUSSION

### Tracking orchid root resupination

We detected the twisting of flowers and resupination of the roots in *E. pusilla*. Flower pedicels bent approximately 30° until the flower opened 126–133 DAG (Figure S3R–S in Appendix S1). By contrast, the leaves of *E. pusilla* did not bend or twist at all during development (Figure S3 in Appendix S1). In this species, the leaves are lanceolate and distichously arranged in rows as in a fan, which physically prevents them from bending or twisting. It is also not necessary, as both sides are equally exposed to light.

The roots of the mature plants were actively resupinating in the first four weeks of scanning, when the CT‐scanned plants still showed a similar development to the controls. From the fifth week of scanning onwards, it took the roots two or more weeks to complete resupination (summarized in Figure S4 in Appendix S1), most likely due to an overexposure to X‐rays. This radiation might have hampered the cell elongation underlying organ twisting (Nakamura and Hashimoto, 2020), which is activated by auxin (Goh, 1983; Mohanty et al., 2012; Panwar et al., 2012). To shed more light on the genetic basis of resupination, a transcriptomic analysis should be carried out. For RNA sequencing, the root tissue of the seedlings should be harvested about seven days before they start to resupinate (or 70 DAG in *E. pusilla*). The optimal harvesting time for the roots of mature plants is 147 DAG.

In this study, the resupination of *E. pusilla* roots could be observed using 3D CT scanning; however, repeated X‐ray radiation exposure clearly delayed the growth and development of the scanned plants. Karahara et al. (2015) and Teramoto et al. (2020) discovered that repeated exposure to X‐ray radiation slows plant development. Like us, these authors found that flowering was delayed or absent when a plant was repeatedly exposed to X‐rays. X‐rays induce the ionization of molecules and the generation of reactive oxygen species (ROS), resulting in oxidative stress (Roldán‐Arjona et al., 2009). The generation of ROS damages biomolecules like pigments, proteins, lipids, carbohydrates, and DNA, damaging the integrity of cells and leading to cell death (Daly, 2012; Das and Roychoudhury, 2014; Wang et al., 2020). Seedlings exposed to X‐rays were less affected in their development than mature plants; therefore, we recommend working with seedlings and immature plants as much as possible when CT scanning live plants over longer periods. In addition, CT scanning with fewer projections could be attempted to reduce the dose per scan.

To further improve the resolution of the scans, we suggest the addition of low concentrations of metal ions into the medium prior to scanning. Six weeks before scanning, the plants should be transferred from this medium into peat moss. Scanning should begin much closer to the onset of the first twisting and/or bending of plant organs to minimize the damage inflicted by long‐term X‐ray exposure. This can be accomplished by examining multiple plants using standard, non‐invasive photography. After obtaining a first estimate of the days when resupination starts, scanning can start much closer to the onset of resupination, thereby minimizing X‐ray‐inflicted damage.

Although it is possible to create a time‐lapse video with the bioinformatic pipeline presented, this requires further development and optimization. Some reconstructions were misaligned (Videos 3 and 4), which may have been caused by the fan‐shaped morphology of *E. pusilla*. The distichously arranged leaves of *E. pusilla* result in a bilaterally flattened plant with identical front and back sides, causing *timelapce.py* to fail to detect which side should be placed in front and vice versa. A second complication was that the images produced in the weekly scans were variable due to the development of new organs, such as leaves and inflorescences, which hampered the reconstructions. Using markers attached to a sample in such a way that the precise orientation can be reconstructed might be one way to reduce misalignment. Alternatively, the sample could be placed on a plastic (X‐ray‐transparent) mount that ensures that it is oriented in the same way each time. Fixed point and virtual rotating have shown excellent results in restoring images with translation and vertical tilt errors during reconstruction (Jun and Yoon, 2017), and so could potentially be successfully applied here. Adding new algorithms to the existing *timelapce.py* script, such as fixed point and virtual rotating, may correct misalignments in future time‐lapse video productions.

### Tracking orchid fruit resupination

*Erycina pusilla* fruits actively bent from 1 to 28 DAP, reaching about 22.5° clockwise in one individual (Figure 4A–D). This is also the period in which the pollen transferred onto the *E. pusilla* flowers developed six pollen tubes and increased in volume. From 56 to 63 DAP, *E. pusilla* fruits twisted at 45° (Figure 4I, J). During the same period, the pollen tubes shrank and fruit cell walls began to lose turgor (Dirks‑Mulder et al., 2019).

Like in *E. pusilla*, *P. equestris* fruits actively moved from 1 to 35 DAP, and continued up to one week before dehiscence (77 DAP). During this time, fruits resupinated from 20° to 180° (see Figure 6). Plant movement is part of growth, and in this case, fruit movement may result from several events during fruit development. Fruit elongation and an increase in the number of cells, which occur 1–35 DAP in *P. equestris* (see Figure S5 and Table S5 in Appendix S1), seem to play a role. Fruit growth and elongation are stimulated by auxin (Devoghalaere et al., 2012); thus, we predict that genes involved in fruit resupination might include ones involved in auxin biosynthesis. Auxin was previously shown to pass basipetally through the gynostemium to the ovaries of orchid flowers, where it jointly regulates resupination alongside other substances produced by the gynostemium or pollinia (Goh et al., 1982; Strauss and Arditti, 1982; Nyman et al., 1985). In *Dendrobium* Sw., auxin was associated with post‐pollination events such as fruit formation (Netlak et al., 2022). Auxin may trigger active movement in early fruit development; for example, its activity in fruit initiation and elongation can cause an imbalance in cell growth, resulting in fruit bending (Moctezuma, 1999). Resupination during fruit ripening might not be correlated with auxin, as this plant hormone inhibits fruit ripening (Liu, 2019). In *Arabidopsis thaliana* (L.) Heynh., the fruit begins to shrink before dehiscence, which is caused by many cells drying out and losing their turgor pressure (Hofhuis et al., 2016). This might also occur in fruits of *E pusilla* and *P. equestris*, and requires further investigation through the implementation of time‐lapse imaging to capture dynamic fruit movements, aiding detailed analysis of timing, extent, and other relevant behaviors. Recommended investigations include exploring physiological mechanisms, hormonal changes, cell growth patterns, and turgor pressure fluctuations during developmental stages. Moreover, conducting transcriptomic analysis and studying auxin's role in post‐pollination resupination may yield valuable insights.

While CT scanning provides insights into plant structures and nuanced movements, acknowledging drawbacks is crucial. The potential impact on proper plant development prompts a critical examination of whether the benefits of CT scanning outweigh those of traditional photography. The meticulous application of CT scanning, with reduced X‐ray exposure, proximity to resupination time, and improved positioning techniques, is expected to enhance precise 3D image reconstruction. This careful approach holds promise for advancing understanding of plant structure while minimizing developmental impacts.

In conclusion, this study highlights orchid root and fruit resupination, emphasizing complexities and potential refinements in imaging techniques. The cautious approach proposed here can serve as a guide for researchers exploring plant movement, contributing to our understanding of plant development.

## AUTHOR CONTRIBUTIONS

D.P., K.J.B., A.K., E.S., and B.G. planned and designed the research. D.P., L.V., and K.D. conducted the experimental work and analyzed and validated the data. L.V. built a bioinformatics pipeline. D.P. and L.V. wrote the original draft of the manuscript. D.P., E.S., and B.G. wrote, reviewed, and edited the manuscript. B.G. supervised the project. All authors approved the final version of the manuscript.

## Supporting information


**Appendix S1**. Supporting tables and figures for “Orchid fruit and root movement analyzed using 2D photographs and a bioinformatics pipeline for processing sequential 3D scans.”

**Appendix S2**. Step‐by‐step guide to scanning and reconstructing images of *Erycina pusilla* plants using SkyScan1172 Micro CT.

## Data Availability

The scripts of the newly built 3D time‐lapse pipeline are available from GitHub (https://github.com/LMVaskimo/3D-Lapse-Pipeline). Reconstruction data are available from Figshare (https://doi.org/10.6084/m9.figshare.24210978): **Data 1.** Reconstruction data for a young *E. pusilla* plant. **Data 2.** Reconstruction data for a mature *E. pusilla* plant.
