# Supplementary material for: Orchid fruit and root movement analyzed using 2D photographs and a bioinformatics pipeline for processing sequential 3D scans
Source: Appl Plant Sci. 2024 Feb 9;12(1):e11567. doi: 10.1002/aps3.11567 (PMC10873816; doi:10.1002/aps3.11567)
Supplement: Supplementary file 1 — Appendix S1. Supporting tables and figures for “Orchid fruit and root movement analyzed using 2D photographs and a bioinformatics pipeline for processing sequential 3D scans.” [file APS3-12-e11567-s002.docx]

**Appendix S1.** Supporting tables and figures for “Orchid fruit and root movement analyzed using 2D photographs and a bioinformatics pipeline for processing sequential 3D scans.”

**Contents**

**Figure S1.** Three-dimensional computed tomography (CT) scan contrast test of the different media in which *Erycina pusilla* was grown.

**Figure S2.** Effect of repeated exposure to X-rays during CT scanning over 19 weeks on development of young, mature, early-flowering, and fully flowering *Erycina pusilla* plants compared with control plants not exposed to any X-rays.

**Figure S3.** Development of *Erycina pusilla* from seed to flowering, recorded in the tissue cultivation laboratory of the Naturalis Biodiversity Center, Leiden, the Netherlands.

**Figure S4.** Resupination of *Erycina pusilla* roots after different exposure times to X-rays.

**Figure S5.** Number of cells of each valve in the fruits of *Phalaenopsis equestris* at eight developmental stages, grown in the tissue cultivation laboratory of the Naturalis Biodiversity Center, Leiden, the Netherlands.

**Table S1.** CT scan protocol for young, mature, early-flowering, and late-flowering *Erycina pusilla* plants*.*

**Table S2.** Response of five *Erycina pusilla* plants exposed to weekly X-ray radiation compared with four control plants not exposed to X-rays.

**Table S3.** Cell numbers in the various *Phalaenopsis equestris* fruit tissues analyzed.

**Table S4.** Area measurements of the various *Phalaenopsis equestris* fruit tissues analyzed. **Table S5.** Main morphological changes observed during the development of *Phalaenopsis equestris* fruits (*N* = 3).

**
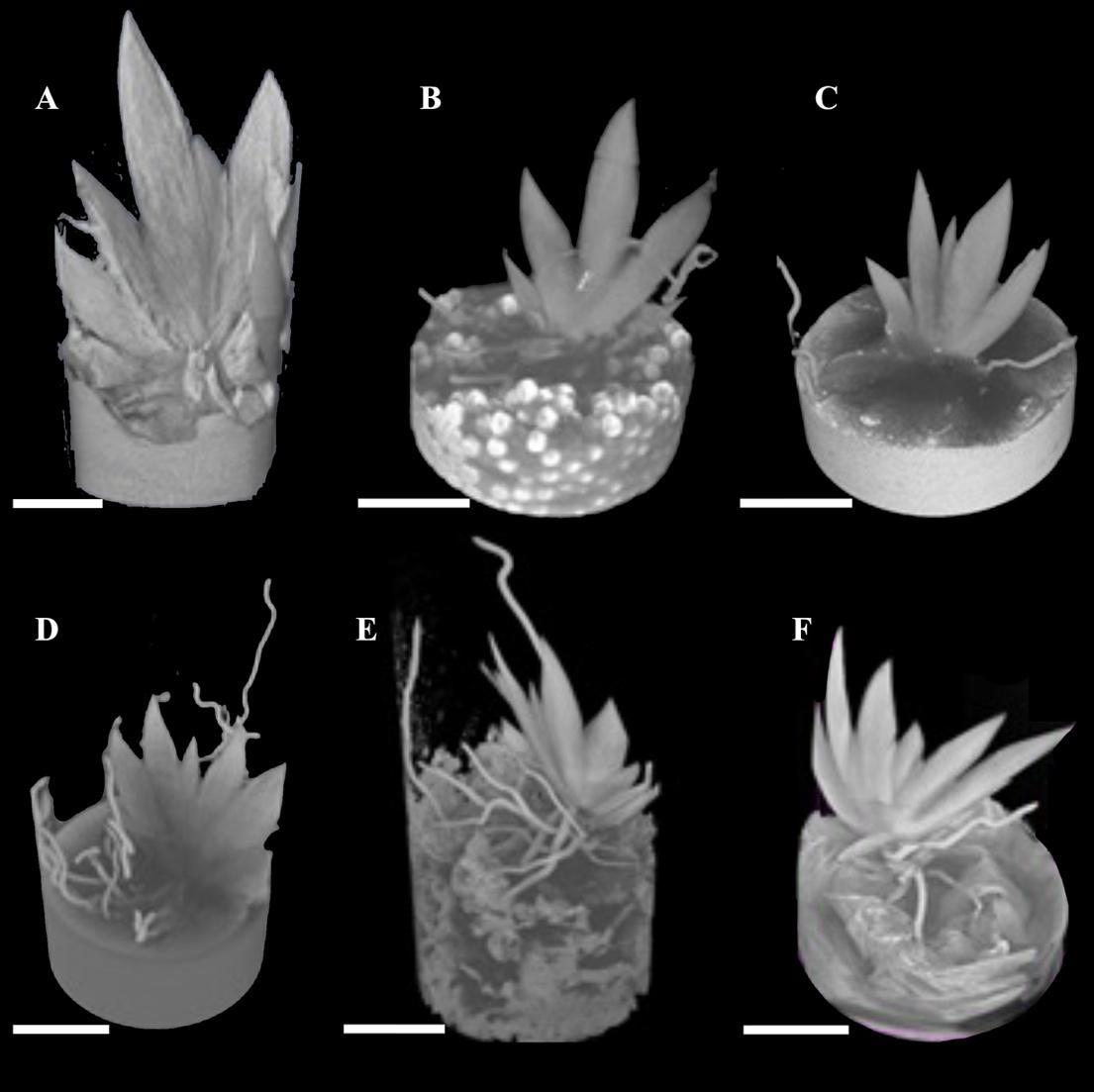
**

**Figure S1.** Three-dimensional computed tomography (CT) scan contrast test of the different media in which *Erycina pusilla* was grown. (A) Gelrite. (B) Glass beads. (C) Sand. (D) Solid agar. (E) *Sphagnum* moss. (F) Cotton. Scale bar = 1 cm.

*
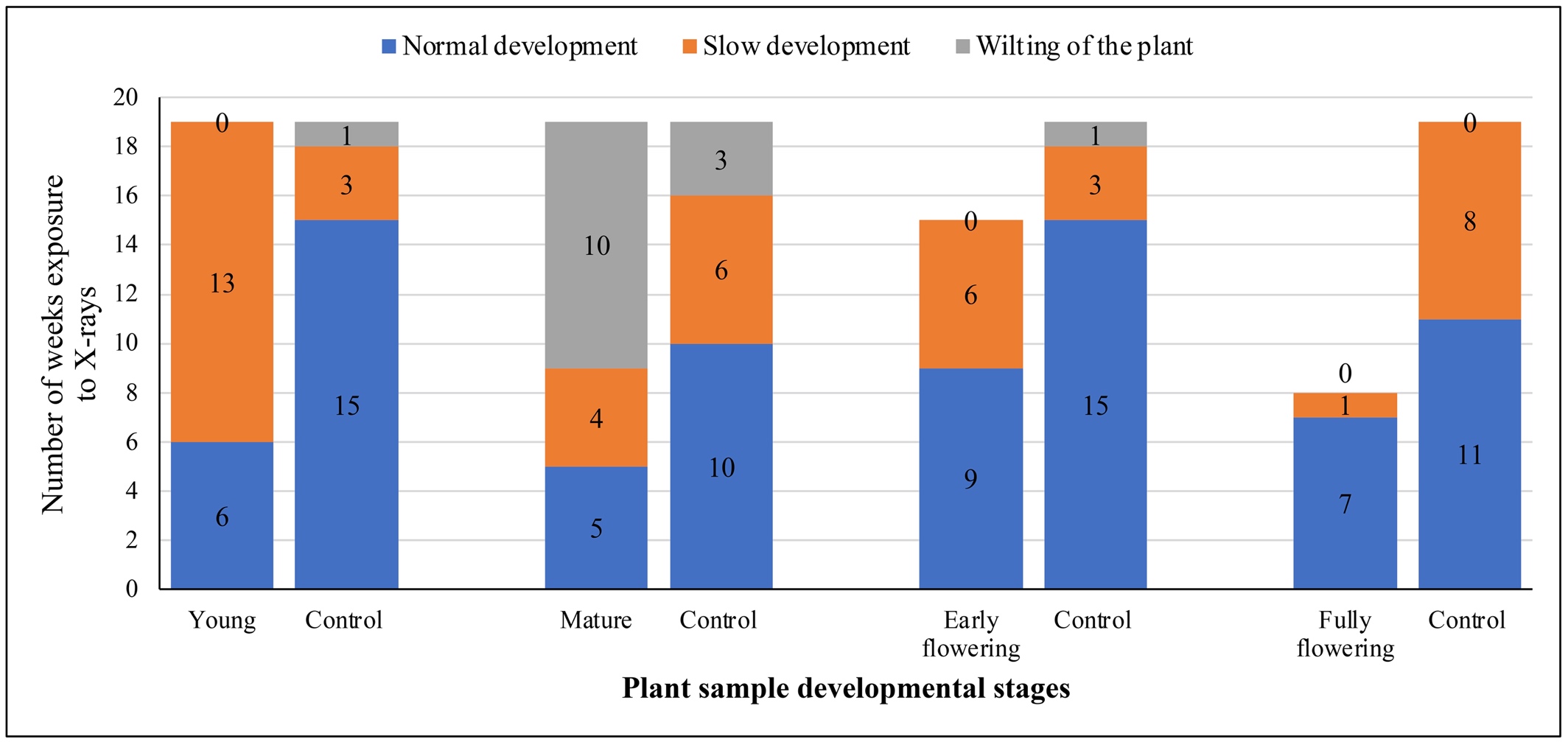
* **Figure S2.** Effect of repeated exposure to X-rays during CT scanning over 19 weeks on development of young, mature, early-flowering, and fully flowering *Erycina pusilla* plants compared with control plants not exposed to any X-rays.


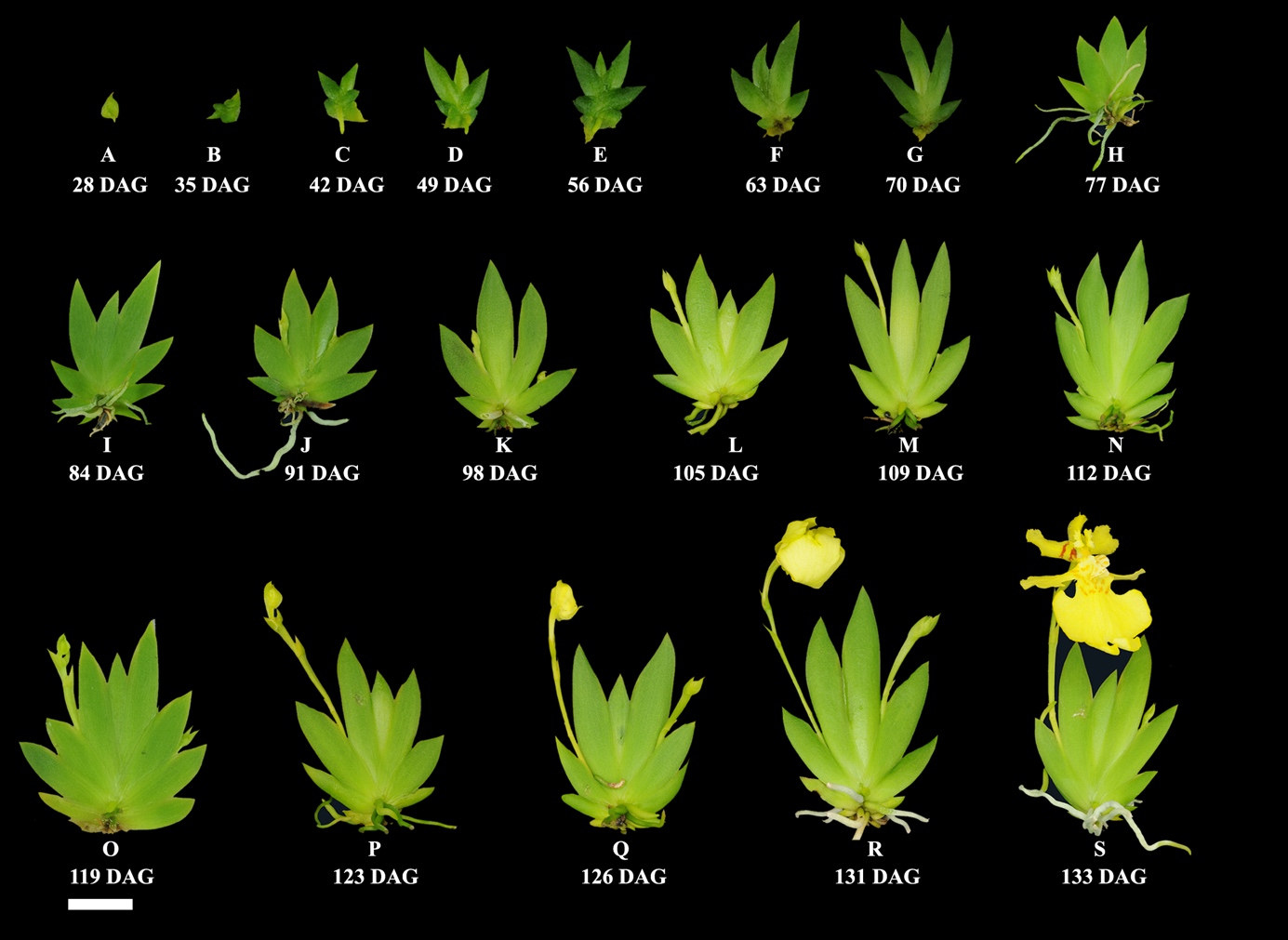


**Figure S3.** Development of *Erycina pusilla* from seed to flowering, recorded in the tissue cultivation laboratory of the Naturalis Biodiversity Center, Leiden, the Netherlands. (A) Leaf-like protocorm visible 28 days after germination (DAG). (B) First development of roots at 35 DAG. (C–G) Seedling development (42–70 DAG). (H, I) Vegetative plant stages (77–84 DAG). (J, K) Inflorescence development and initiation of resupination ±180° of the flower (91–98 DAG). (L–N) Elongation of the inflorescence stalk (105–112 DAG). (O) Floral bud emerging from the bract (119 DAG). (P, Q) 30° bending of the pedicel until the flower opens (123–126 DAG). (R, S) Flower opening (131–133 DAG). Scale bar = 1 cm.


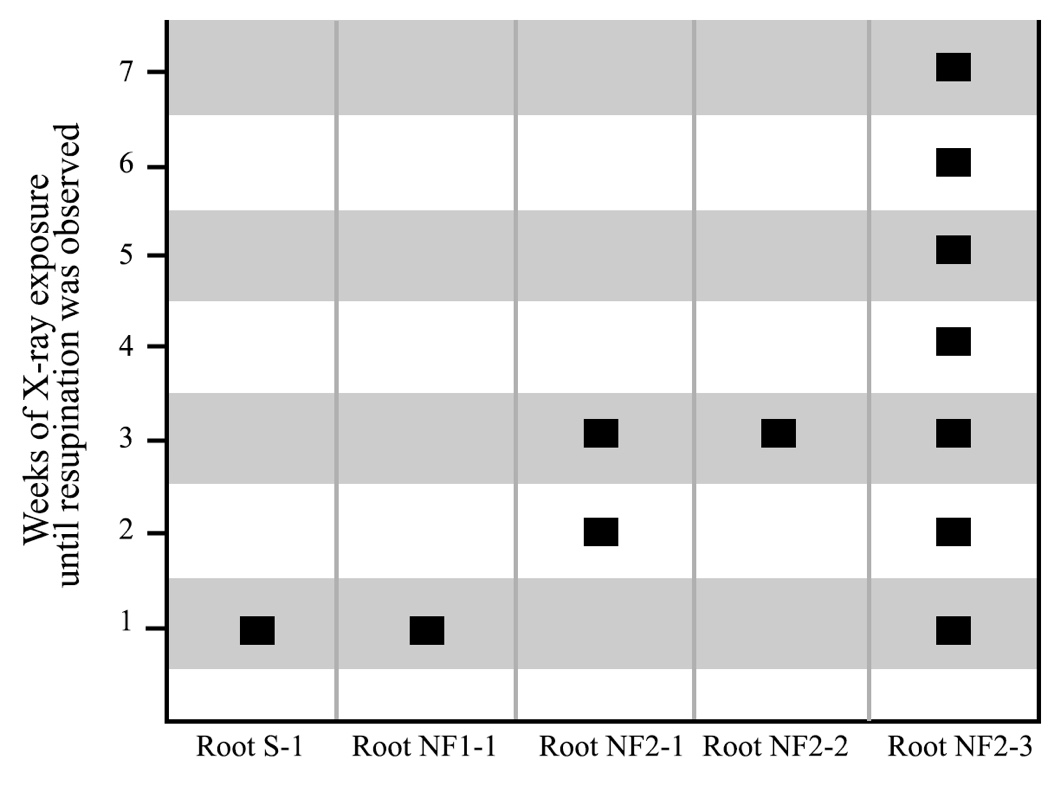


**Figure S4.** Resupination of *Erycina pusilla* roots after different exposure times to X-rays. Root S-1 = root of seedling; Root NF1-1 = root of the first non-flowering plant replicate; Root NF2-1 = first root of the second non-flowering plant replicate; Root NF2-2 = second root of the second non-flowering plant replicate; Root NF2-3 = third root of the second non-flowering plant replicate.


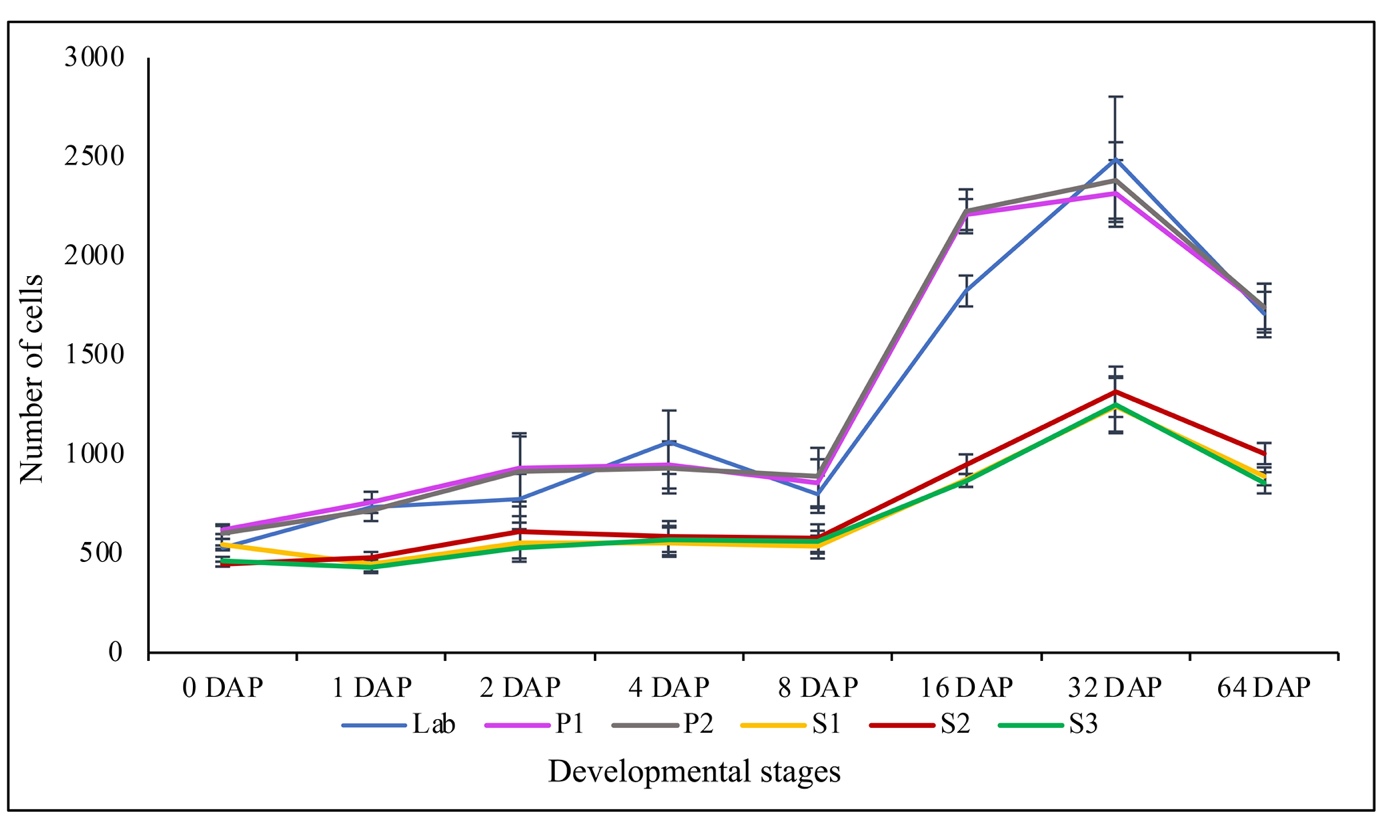


**Figure S5.** Number of cells of each valve in the fruits of *Phalaenopsis equestris* at eight developmental stages, grown in the tissue cultivation laboratory of the Naturalis Biodiversity Center, Leiden, the Netherlands. DAP = days after pollination; Lab = fertile valve derived from the labellum; P1 = fertile valve derived from the first petal; P2 = fertile valve derived from the second petal; S1 = sterile valve derived from the first lateral sepal; S2 = sterile valve originated from dorsal sepal; S3 = sterile valve derived from the second lateral sepal. Error bars are indicated in black.

**Table S1.** CT scan protocol for young, mature, early-flowering, and late-flowering *Erycina pusilla* plants*.*

| **Features** | **Young plant** | **Mature plant** | **Early flowering** | **Late flowering** |
| --- | --- | --- | --- | --- |
| Lens magnitude | 0× | 0× | 0× | 0× |
| Filter | None | None | None | None |
| Acceleration voltage/power (kV/mA) | 30/175 | 40/250 | 35/217 | 35/217 |
| Rotation step | 0.40 | 0.40 | 0.40 | 0.40 |
| Projections | 1400 | 1400 | 1400 | 1400 |
| Exposure time (ms) | 700 | 675 | 685 | 685 |

**Table S2.** Response of five *Erycina pusilla* plants exposed to weekly X-ray radiation compared with four control plants not exposed to X-rays.

| **Organ/**  **Sample** | **Young seedling** | **Mature plant 1** | **Mature plant 2** | **Mature plant 3** | **Late-flowering plant** | **Control plant 1** | **Control plant 2** | **Control plant 3** | **Control plant 4** |
| --- | --- | --- | --- | --- | --- | --- | --- | --- | --- |
| Roots | Present before | Present before | Present before | Present before | Present before | Present before | Present before | Present before | Present before |
| Leaves | Present before | Present before | Present before | Present before | Present before | Present before | Present before | Present before | Present before |
| Inflorescence | Absent | Absent | Absent | Absent | Present before | Further developed | Further developed | Further developed | Further developed |
| Flowers | Absent | Absent | Absent | Absent | Absent | Further developed | Absent | Absent | Further developed |
| Fruits | Absent | Absent | Absent | Absent | Absent | Absent | Absent | Absent | Absent |

**Table S3.** Total cell numbers in the various *Phalaenopsis equestris* fruit tissues analyzed.

| **Stages** | **Biological replicates** | **Technical replicates** | **Number of cells** | | | | | | | | |
| --- | --- | --- | --- | --- | --- | --- | --- | --- | --- | --- | --- |
|  |  |  | **Labellum** | **Petal 1** | **Petal 2** | **Sepal 1** | **Sepal 2** | **Sepal 3** | **Fertile valve** | **Sterile valve** | **Total cells** |
| 0 DAP | 1 | 1 | 537 | 622 | 619 | 564 | 450 | 470 | 1778 | 1484 | 3262 |
|  | 1 | 2 | 514.5 | 557 | 567 | 524 | 437 | 406.5 | 1638 | 1367 | 3005 |
|  | 1 | 3 | 541 | 691 | 636 | 550 | 463.5 | 507.5 | 1867 | 1521 | 3388 |
|  | 2 | 1 | 524 | 656 | 644 | 656 | 475 | 482 | 1824 | 1613 | 3437 |
|  | 2 | 2 | 529 | 631 | 681 | 652 | 498 | 440 | 1841 | 1590 | 3431 |
|  | 2 | 3 | 572 | 772 | 760 | 594 | 499 | 608 | 2104 | 1701 | 3805 |
|  | 3 | 1 | 550 | 588 | 594 | 472 | 425 | 458 | 1732 | 1355 | 3087 |
|  | 3 | 2 | 500 | 483 | 452 | 395 | 376 | 373 | 1435 | 1144 | 2579 |
|  | 3 | 3 | 510 | 609 | 511 | 506 | 428 | 407 | 1630 | 1341 | 2971 |
| 1 DAP | 1 | 1 | 618 | 614 | 463 | 236 | 332 | 322 | 1695 | 890 | 2585 |
|  | 1 | 2 | 676 | 669 | 591 | 351 | 395 | 366 | 1936 | 1112 | 3048 |
|  | 1 | 3 | 622 | 590 | 588 | 425 | 423 | 348 | 1800 | 1196 | 2996 |
|  | 2 | 1 | 760 | 785 | 815 | 599 | 595 | 597 | 2360 | 1791 | 4151 |
|  | 2 | 2 | 827 | 762 | 768 | 595 | 604 | 587 | 2357 | 1786 | 4143 |
|  | 2 | 3 | 676 | 559 | 598 | 470 | 483 | 460 | 1833 | 1413 | 3246 |
|  | 3 | 1 | 858 | 999 | 909 | 470 | 509 | 456 | 2766 | 1435 | 4201 |
|  | 3 | 2 | 756 | 892 | 858 | 426 | 474 | 390 | 2506 | 1290 | 3796 |
|  | 3 | 3 | 788 | 941 | 861 | 455 | 497 | 384 | 2590 | 1336 | 3926 |
| 2 DAP | 1 | 1 | 447 | 441 | 477 | 380 | 407 | 308 | 1365 | 1095 | 2460 |
|  | 1 | 2 | 527 | 539 | 513 | 308 | 403 | 307 | 1579 | 1018 | 2597 |
|  | 1 | 3 | 521 | 434 | 456 | 309 | 378 | 317 | 1411 | 1004 | 2415 |
|  | 2 | 1 | 1185 | 1585 | 1621 | 829 | 932 | 793 | 4391 | 2554 | 6945 |
|  | 2 | 2 | 1278 | 1614 | 1627 | 838 | 896 | 810 | 4519 | 2544 | 7063 |
|  | 2 | 3 | 1317 | 1602 | 1571 | 820 | 918 | 786 | 4490 | 2524 | 7014 |
|  | 3 | 1 | 598 | 626 | 580 | 494 | 420 | 424 | 1804 | 1338 | 3142 |
|  | 3 | 2 | 628 | 841 | 810 | 579 | 637 | 585 | 2279 | 1801 | 4080 |
|  | 3 | 3 | 511 | 730 | 603 | 400 | 509 | 472 | 1844 | 1381 | 3225 |
| 4 DAP | 1 | 1 | 1043 | 927 | 759 | 452 | 464 | 482 | 2729 | 1398 | 4127 |
|  | 1 | 2 | 852 | 674 | 663 | 422 | 365 | 400 | 2189 | 1187 | 3376 |
|  | 1 | 3 | 863 | 663 | 650 | 440 | 416 | 449 | 2176 | 1305 | 3481 |
|  | 2 | 1 | 740 | 767 | 788 | 469 | 433 | 449 | 2295 | 1351 | 3646 |
|  | 2 | 2 | 541 | 646 | 700 | 323 | 477 | 377 | 1887 | 1177 | 3064 |
|  | 2 | 3 | 570 | 649 | 618 | 385 | 466 | 394 | 1837 | 1245 | 3082 |
|  | 3 | 1 | 1728 | 1283 | 1330 | 841 | 876 | 880 | 4341 | 2597 | 6938 |
|  | 3 | 2 | 1651 | 1361 | 1613 | 820 | 876 | 848 | 4625 | 2544 | 7169 |
|  | 3 | 3 | 1581 | 1567 | 1230 | 869 | 899 | 833 | 4378 | 2601 | 6979 |
| 8 DAP | 1 | 1 | 369 | 460 | 455 | 281 | 282 | 313 | 1284 | 876 | 2160 |
|  | 1 | 2 | 437 | 411 | 321 | 264 | 319 | 355 | 1169 | 938 | 2107 |
|  | 1 | 3 | 471 | 340 | 270 | 418 | 338 | 332 | 1081 | 1088 | 2169 |
|  | 2 | 1 | 1017 | 1300 | 1466 | 589 | 781 | 686 | 3783 | 2056 | 5839 |
|  | 2 | 2 | 962 | 1305 | 1310 | 616 | 794 | 701 | 3577 | 2111 | 5688 |
|  | 2 | 3 | 877 | 1128 | 1201 | 653 | 728 | 588 | 3206 | 1969 | 5175 |
|  | 3 | 1 | 962 | 932 | 944 | 635 | 636 | 672 | 2838 | 1943 | 4781 |
|  | 3 | 2 | 940 | 948 | 962 | 647 | 636 | 700 | 2850 | 1983 | 4833 |
|  | 3 | 3 | 1162 | 871 | 1075 | 703 | 677 | 691 | 3108 | 2071 | 5179 |
| 16 DAP | 1 | 1 | 1575 | 2337 | 2202 | 898 | 1013 | 987 | 6114 | 2898 | 9012 |
|  | 1 | 2 | 2325 | 2646 | 2913 | 861 | 1237 | 840 | 7884 | 2938 | 10822 |
|  | 1 | 3 | 2013 | 2209 | 2570 | 803 | 1075 | 837 | 6792 | 2715 | 9507 |
|  | 2 | 1 | 1875 | 2162 | 2109 | 976 | 846 | 950 | 6146 | 2772 | 8918 |
|  | 2 | 2 | 1889 | 2405 | 2266 | 810 | 845 | 993 | 6560 | 2648 | 9208 |
|  | 2 | 3 | 1858 | 2207 | 1913 | 940 | 975 | 850 | 5978 | 2765 | 8743 |
|  | 3 | 1 | 1667 | 1865 | 1879 | 694 | 788 | 807 | 5411 | 2289 | 7700 |
|  | 3 | 2 | 1610 | 1945 | 2100 | 902 | 825 | 702 | 5655 | 2429 | 8084 |
|  | 3 | 3 | 1620 | 2127 | 2108 | 958 | 965 | 852 | 5855 | 2775 | 8630 |
| 32 DAP | 1 | 1 | 1414 | 1785 | 1731 | 787 | 883 | 728 | 4930 | 2398 | 7328 |
|  | 1 | 2 | 1551 | 1777 | 1700 | 740 | 784 | 738 | 5028 | 2262 | 7290 |
|  | 1 | 3 | 1453 | 1657 | 1685 | 663 | 794 | 692 | 4795 | 2149 | 6944 |
|  | 2 | 1 | 3646 | 2919 | 2912 | 1439 | 1560 | 1816 | 9477 | 4815 | 14292 |
|  | 2 | 2 | 3649 | 2930 | 2890 | 1754 | 1463 | 1589 | 9469 | 4806 | 14275 |
|  | 2 | 3 | 3622 | 2820 | 3222 | 1743 | 1486 | 1472 | 9664 | 4701 | 14365 |
|  | 3 | 1 | 2476 | 2497 | 2546 | 1489 | 1739 | 1365 | 7519 | 4593 | 12112 |
|  | 3 | 2 | 2116 | 2176 | 2334 | 1255 | 1548 | 1458 | 6626 | 4261 | 10887 |
|  | 3 | 3 | 2486 | 2323 | 2411 | 1330 | 1607 | 1422 | 7220 | 4359 | 11579 |
| 64 DAP | 1 | 1 | 2145 | 2307 | 2226 | 1090 | 1217 | 1083 | 6678 | 3390 | 10068 |
|  | 1 | 2 | 2065 | 2182 | 2185 | 731 | 743 | 744 | 6432 | 2218 | 8650 |
|  | 1 | 3 | 2081 | 2123 | 2127 | 672 | 834 | 705 | 6331 | 2211 | 8542 |
|  | 2 | 1 | 1850 | 1669 | 1801 | 1046 | 1200 | 1052 | 5320 | 3298 | 8618 |
|  | 2 | 2 | 1619 | 1632 | 1604 | 1000 | 1022 | 956 | 4855 | 2978 | 7833 |
|  | 2 | 3 | 1558 | 1346 | 1484 | 952 | 1013 | 958 | 4388 | 2923 | 7311 |
|  | 3 | 1 | 1250 | 1443 | 1408 | 885 | 965 | 713 | 4101 | 2563 | 6664 |
|  | 3 | 2 | 1516 | 1439 | 1506 | 854 | 1016 | 793 | 4461 | 2663 | 7124 |
|  | 3 | 3 | 1286 | 1519 | 1371 | 786 | 1049 | 717 | 4176 | 2552 | 6728 |

Note: DAP = days after pollination.

**Table S4.** Area measurements of the various *Phalaenopsis equestris* fruit tissues analyzed.

| **Stages** | **Biological replicates** | **Technical replicates** | **Area (mm²)** | | | | | | | |
| --- | --- | --- | --- | --- | --- | --- | --- | --- | --- | --- |
|  |  |  | **Total fruit** | **Labellum** | **Petal 1** | **Petal 2** | **Sepal 1** | **Sepal 2** | **Sepal 3** | **Total area valves** |
| 0 DAP | 1 | 1 | 1.248 | 0.1865 | 0.226 | 0.256 | 0.17 | 0.21 | 0.162 | 1.217 |
|  | 1 | 2 | 1.237 | 0.198 | 0.217 | 0.188 | 0.18 | 0.21 | 0.22 | 1.215 |
|  | 1 | 3 | 1.242 | 0.1925 | 0.245 | 0.249 | 0.2 | 0.16 | 0.182 | 1.228 |
|  | 2 | 1 | 1.580 | 0.231 | 0.270 | 0.336 | 0.203 | 0.329 | 0.194 | 1.563 |
|  | 2 | 2 | 1.567 | 0.249 | 0.260 | 0.197 | 0.218 | 0.322 | 0.309 | 1.555 |
|  | 2 | 3 | 1.583 | 0.241 | 0.318 | 0.322 | 0.265 | 0.202 | 0.228 | 1.576 |
|  | 3 | 1 | 0.915 | 0.142 | 0.181 | 0.175 | 0.144 | 0.100 | 0.129 | 0.871 |
|  | 3 | 2 | 0.906 | 0.147 | 0.174 | 0.178 | 0.141 | 0.103 | 0.131 | 0.874 |
|  | 3 | 3 | 0.900 | 0.144 | 0.172 | 0.176 | 0.144 | 0.109 | 0.135 | 0.88 |
| 1 DAP | 1 | 1 | 1.376 | 0.355 | 0.281 | 0.263 | 0.180 | 0.141 | 0.177 | 1.397 |
|  | 1 | 2 | 1.387 | 0.366 | 0.307 | 0.277 | 0.180 | 0.145 | 0.210 | 1.485 |
|  | 1 | 3 | 1.461 | 0.277 | 0.268 | 0.311 | 0.235 | 0.107 | 0.212 | 1.41 |
|  | 2 | 1 | 1.201 | 0.207 | 0.224 | 0.240 | 0.173 | 0.157 | 0.160 | 1.161 |
|  | 2 | 2 | 1.174 | 0.206 | 0.214 | 0.226 | 0.176 | 0.173 | 0.147 | 1.142 |
|  | 2 | 3 | 1.168 | 0.202 | 0.222 | 0.225 | 0.164 | 0.157 | 0.153 | 1.123 |
|  | 3 | 1 | 0.908 | 0.162 | 0.229 | 0.219 | 0.096 | 0.092 | 0.085 | 0.883 |
|  | 3 | 2 | 0.901 | 0.158 | 0.213 | 0.210 | 0.09 | 0.09 | 0.081 | 0.841 |
|  | 3 | 3 | 0.898 | 0.165 | 0.216 | 0.212 | 0.095 | 0.087 | 0.082 | 0.857 |
| 2 DAP | 1 | 1 | 1.269 | 0.197 | 0.190 | 0.244 | 0.189 | 0.206 | 0.205 | 1.231 |
|  | 1 | 2 | 1.345 | 0.246 | 0.262 | 0.253 | 0.202 | 0.225 | 0.161 | 1.349 |
|  | 1 | 3 | 1.307 | 0.222 | 0.226 | 0.249 | 0.196 | 0.216 | 0.183 | 1.29 |
|  | 2 | 1 | 1.983 | 0.306 | 0.469 | 0.426 | 0.215 | 0.279 | 0.214 | 1.909 |
|  | 2 | 2 | 1.971 | 0.307 | 0.467 | 0.442 | 0.213 | 0.258 | 0.213 | 1.9 |
|  | 2 | 3 | 1.960 | 0.308 | 0.469 | 0.439 | 0.215 | 0.266 | 0.200 | 1.897 |
|  | 3 | 1 | 1.038 | 0.176 | 0.215 | 0.193 | 0.139 | 0.130 | 0.144 | 0.997 |
|  | 3 | 2 | 1.001 | 0.161 | 0.201 | 0.192 | 0.136 | 0.133 | 0.139 | 0.962 |
|  | 3 | 3 | 1.027 | 0.166 | 0.212 | 0.189 | 0.139 | 0.140 | 0.143 | 0.989 |
| 4 DAP | 1 | 1 | 1.396 | 0.327 | 0.270 | 0.293 | 0.182 | 0.155 | 0.185 | 1.412 |
|  | 1 | 2 | 1.335 | 0.270 | 0.296 | 0.302 | 0.191 | 0.125 | 0.159 | 1.343 |
|  | 1 | 3 | 1.343 | 0.268 | 0.328 | 0.271 | 0.18 | 0.14 | 0.157 | 1.343 |
|  | 2 | 1 | 1.257 | 0.194 | 0.283 | 0.287 | 0.150 | 0.158 | 0.125 | 1.197 |
|  | 2 | 2 | 1.257 | 0.190 | 0.282 | 0.291 | 0.126 | 0.168 | 0.151 | 1.208 |
|  | 2 | 3 | 1.275 | 0.187 | 0.281 | 0.278 | 0.133 | 0.177 | 0.154 | 1.21 |
|  | 3 | 1 | 2.040 | 0.340 | 0.443 | 0.502 | 0.232 | 0.221 | 0.215 | 1.953 |
|  | 3 | 2 | 2.050 | 0.342 | 0.459 | 0.485 | 0.234 | 0.206 | 0.213 | 1.939 |
|  | 3 | 3 | 2.058 | 0.336 | 0.500 | 0.467 | 0.209 | 0.214 | 0.221 | 1.947 |
| 8 DAP | 1 | 1 | 2.062 | 0.379 | 0.458 | 0.388 | 0.306 | 0.253 | 0.278 | 2.062 |
|  | 1 | 2 | 1.927 | 0.391 | 0.301 | 0.317 | 0.25 | 0.31 | 0.244 | 1.813 |
|  | 1 | 3 | 1.685 | 0.401 | 0.256 | 0.262 | 0.23 | 0.26 | 0.295 | 1.694 |
|  | 2 | 1 | 2.238 | 0.509 | 0.317 | 0.462 | 0.247 | 0.238 | 0.336 | 2.109 |
|  | 2 | 2 | 2.262 | 0.515 | 0.335 | 0.483 | 0.253 | 0.243 | 0.295 | 2.124 |
|  | 2 | 3 | 2.281 | 0.530 | 0.325 | 0.465 | 0.256 | 0.262 | 0.325 | 2.163 |
|  | 3 | 1 | 2.786 | 0.446 | 0.556 | 0.535 | 0.328 | 0.333 | 0.316 | 2.514 |
|  | 3 | 2 | 2.870 | 0.460 | 0.537 | 0.558 | 0.306 | 0.350 | 0.347 | 2.558 |
|  | 3 | 3 | 2.733 | 0.441 | 0.515 | 0.538 | 0.308 | 0.327 | 0.324 | 2.453 |
| 16 DAP | 1 | 1 | 8.853 | 1.254 | 2.148 | 2.167 | 0.548 | 0.864 | 0.559 | 7.54 |
|  | 1 | 2 | 8.536 | 1.305 | 1.976 | 1.956 | 0.496 | 0.798 | 0.564 | 7.095 |
|  | 1 | 3 | 8.650 | 1.323 | 1.970 | 1.929 | 0.476 | 0.818 | 0.583 | 7.099 |
|  | 2 | 1 | 9.328 | 1.427 | 1.992 | 2.218 | 0.728 | 0.782 | 0.667 | 7.814 |
|  | 2 | 2 | 9.294 | 1.410 | 2.222 | 1.979 | 0.673 | 0.797 | 0.730 | 7.811 |
|  | 2 | 3 | 9.560 | 1.467 | 2.236 | 2.012 | 0.728 | 0.820 | 0.717 | 7.98 |
|  | 3 | 1 | 10.275 | 1.484 | 2.363 | 2.371 | 0.872 | 0.959 | 0.721 | 8.77 |
|  | 3 | 2 | 10.289 | 1.453 | 2.384 | 2.382 | 0.875 | 0.943 | 0.730 | 8.767 |
|  | 3 | 3 | 10.257 | 1.444 | 2.374 | 2.370 | 0.859 | 0.962 | 0.711 | 8.72 |
| 32 DAP | 1 | 1 | 10.668 | 1.796 | 2.621 | 2.586 | 0.698 | 1.035 | 0.690 | 9.426 |
|  | 1 | 2 | 10.739 | 1.806 | 2.578 | 2.582 | 0.711 | 1.027 | 0.673 | 9.377 |
|  | 1 | 3 | 10.772 | 1.794 | 2.521 | 2.571 | 0.675 | 1.025 | 0.706 | 9.292 |
|  | 2 | 1 | 30.530 | 6.382 | 4.835 | 5.879 | 1.945 | 1.858 | 2.237 | 23.14 |
|  | 2 | 2 | 30.413 | 4.869 | 6.426 | 5.776 | 1.917 | 2.268 | 1.910 | 23.17 |
|  | 2 | 3 | 29.912 | 4.587 | 5.716 | 6.122 | 1.894 | 2.236 | 1.787 | 22.34 |
|  | 3 | 1 | 24.920 | 4.050 | 4.410 | 4.307 | 1.642 | 2.370 | 1.615 | 18.39 |
|  | 3 | 2 | 24.437 | 3.895 | 4.106 | 4.449 | 1.671 | 2.321 | 1.800 | 18.24 |
|  | 3 | 3 | 24.590 | 3.946 | 4.290 | 4.287 | 1.650 | 2.448 | 1.628 | 18.25 |
| 64 DAP | 1 | 1 | 26.902 | 4.564 | 5.829 | 5.409 | 1.717 | 2.378 | 1.601 | 21.5 |
|  | 1 | 2 | 27.234 | 4.537 | 5.320 | 6.007 | 1.575 | 2.413 | 1.657 | 21.51 |
|  | 1 | 3 | 27.573 | 4.515 | 6.149 | 5.502 | 1.773 | 2.586 | 1.568 | 22.09 |
|  | 2 | 1 | 37.376 | 5.877 | 5.735 | 6.432 | 2.500 | 3.130 | 2.680 | 26.35 |
|  | 2 | 2 | 37.369 | 5.742 | 6.238 | 5.971 | 2.708 | 3.154 | 2.448 | 26.26 |
|  | 2 | 3 | 38.685 | 5.824 | 6.645 | 6.090 | 2.705 | 3.250 | 2.471 | 26.99 |
|  | 3 | 1 | 45.078 | 6.545 | 8.139 | 7.753 | 2.513 | 3.574 | 1.912 | 30.44 |
|  | 3 | 2 | 44.726 | 6.476 | 8.020 | 7.877 | 2.474 | 3.683 | 1.921 | 30.45 |
|  | 3 | 3 | 44.603 | 6.389 | 8.197 | 7.746 | 2.437 | 3.647 | 1.886 | 30.3 |

Note: DAP = days after pollination.

**Table S5**. Main morphological changes observed during the development of *Phalaenopsis equestris* fruits (*N* = 3).

| **Period (DAP)** | **Main morphological changes** |
| --- | --- |
| 0–8 | Elongation of fruit |
|  | Cell division and growth in fertile and sterile valves |
|  | Increase in volume of fruit |
| 8–16 | Development of six pollen tube bundles |
|  | Formation of dehiscence zones |
|  | Elongation of fruit |
|  | Cell division and growth in fertile and sterile valves |
|  | Increase in volume of fruit |
| 16–32 | Shrinking of pollen tube bundles |
|  | Trichome development |
|  | Formation of dehiscence zones |
|  | Elongation of fruit |
|  | Cell division and growth in fertile and sterile valves |
|  | Increase in volume of fruit |
| 32–64 | Increase of volume of cells in sterile and fertile valves |
|  | Disappearance of pollen tube bundles |
|  | Increase in volume of fruit |
| 64–154 | Shrinking of fruit |
|  | Seed maturation |
| 154–168 | Dehiscence |

Note: DAP = days after pollination.
