# Supplementary material for: Orchid fruit and root movement analyzed using 2D photographs and a bioinformatics pipeline for processing sequential 3D scans
Source: Appl Plant Sci. 2024 Feb 9;12(1):e11567. doi: 10.1002/aps3.11567 (PMC10873816; doi:10.1002/aps3.11567)
Supplement: Supplementary file 2 — Appendix S2. Step‐by‐step guide to scanning and reconstructing images of Erycina pusilla plants using SkyScan1172 Micro CT. [file APS3-12-e11567-s001.docx]

**Appendix S2**. Step-by-step guide to scanning and reconstructing images of *Erycina pusilla* plants using SkyScan1172 Micro CT.

- - - 1. **Before scanning:**

1. *Erycina pusilla* plants were cultured in sterile sphagnum moss with 10 mL of liquid orchid maintenance medium (see main text, section “Tissue culture of *Erycina pusilla*”). The plantlets were placed in a climate chamber. All *E. pusilla* plant samples were scanned once a week.
2. Start PC and/or scanner (if necessary). The scanner can be started by turning the key on the right side of the MicroCT SkyScan (Bruker, Billerica, Massachusetts, USA). Start the SkyScan1172 software.
3. Attach the container to the copper/gold-colored round holder and close using the soldering iron.
4. To attach the container, use the yellow wax in the pot. Melt the wax until it turns to liquid, and place the container in the middle of the holder.
5. **SkyScan1172 Software usage**
6. After starting the program, hit the hazard symbol to start the X-ray. If the scanner has been off for 8 h or more, it must warm up first. This takes about 15 min. The camera or TV can also be used to start the warming-up process (these both also start the X-ray). All previously mentioned symbols are found at the top toolbar of the software.
7. After the warm-up, click the new triangular hazard symbol (on the right of the screen). A new screen should pop up, in which the voltage can be changed. Slide the bar to the desired voltage and click *Apply*. The desired voltages are found in Table S1 (Appendix S1)*.*
8. The camera should be set to the medium setting. This can be done by clicking the square with four arrows pointing outward and selecting the medium setting.
9. **Taking a flat field reference**
   1. Before scanning, a flat field reference must be taken. Prior to this, the flat field correction must first be turned off. This can be accomplished by going to *Options* in the toolbar at the top and selecting *Preferences*. Here, deselect both the TV and camera options for the flat field correction and click *Apply*.
   2. Next, the flat field can be referenced by going to *Options* and selecting *Acquisition modes*. If this option is unavailable, hit *ctrl+alt+shift+s* and try again.
   3. The Acquisition modes screen pops up. Here, the desired recipe should be entered. The exposure time should be entered at the top, where the ‘->’ is pointing (most probably somewhere on the top left). This differs from how the scan is set but will be the same for all scans on the *Erycina pusilla*. The value can be changed by pressing the arrow keys next to the value (it only goes per 5).
   4. Below the exposure times, the voltage and watts should be entered.
   5. The voltage and watts are provided in Table S1 (Appendix S1) and should correspond to the values on the voltage-changing screen.
   6. After entering the recipe described above, select *Acquire bright + dark for current mode* and click *Apply* to make the flat field reference.
   7. The X-ray is turned off during the flat field reference, and all values should be restored afterward. However, this usually fails, as it returns the values to 60 instead of the desired voltage. If this is the case, change it back to the desired voltage using the slider and perform a new flat field reference as described above.
   8. If the flat field was done correctly, turn the flat field correction on again for both the TV and the camera in *Options* -> *Preferences.*
10. **Placing the plant in the scanner**
    1. After performing the flat field reference, the sample must be placed inside the scanner. The door to the scanner can be opened by pressing the door icon on the top toolbar. After this, the slider door will open, and the sample, including the holder, can be placed into the scanner. To avoid the sample moving during the scan, ensure it is tightened.
    2. Close the door by pressing the door icon at the top of the toolbar again.
    3. Now, the X-ray can be started by pressing the TV icon, and the plant image should appear.
    4. A live video image of the plant can be obtained by pressing the lamp icon on the top toolbar. It will first give an error; press close and select *Options* on the popped-up screen. Change the camera number from 3 to 2 and close. Now, a live image of the plant should be visible.
11. **Starting the scan**
    1. Before starting the scan, check whether the flat field reference was performed correctly. This can be done by right clicking the image. Several values and lines will appear, and the flat field is correct when the minimum is between 20% and 40%. If not, the flat field should be redone (please remember that the plant must be removed from the scanner first!).
    2. First, make a new folder in the correct data folder. Name and number them as the previous scans have been numbered.
    3. To start the scan, go to *Actions* in the toolbar at the top, and then select *Set oversized scan*.
    4. A new screen should pop up where the scan can be started. First, press the *Scout scan* button. This will move the plant to the bottom and slowly lift it. When the container is in the field of view on the *Set oversize scan* window, press *Interrupt*. The movement of the plant and, thus, the timing of the interruption can be nicely followed using the live video footage, which was previously activated.
    5. Now, the correct scan name must be entered in the *prefix* box. Please note the underscore at the end, which is obligatory.
    6. After entering the scan name, the scan region must be selected. This is done by pressing the *Top* button and selecting the top of the scan. The same goes for the bottom of the region by choosing the *Bottom* button and selecting the bottom. For all scans except the small plant, the top is the border of the container’s cap and the container (just below the lid), and the bottom is the bottom of the container. The small plant scan takes the halves of these regions, so the top is half of the air part of the container and the bottom half of the medium.
    7. Next, press *Start scan*. A new screen will pop up. The only thing that needs to be changed on this screen is the destination of the scans. This can be done by pressing *Browse* and going to the folder destination.
    8. Press *Start scan*. The scan now starts to run.
12. **After the scan**
    1. The oversized scan screen can be closed, and the plant can be removed by pressing the door button to open the scanner door. The door can be closed again using the door button.
    2. If another scan must be done, repeat steps 2, 4, and 5.
    3. Reconstruction can be made, but this is not of utmost importance, as they can always be made later and the scans are of higher priority at this time. A detailed guide on how to start the reconstruction can be found in Table S1 (Appendix S1).
13. **Image reconstruction process**
14. The reconstruction software used is NRecon (Carl Zeiss, Oberkochen, Germany). Open the software and wait for the software to acquire a connection. The pop-up screen can be moved to the left, as it has to stay open for the entire reconstruction duration.
15. After gaining a connection, a screen to open data pops up. Go to the preferred scan data previously obtained. Scroll somewhat down in the scan folder, randomly select an image, and click open.
16. When the scan has been loaded, it will show a preview on the screen*.* The only thing that must be done is setting the destination folder.
17. Go to the *Output* menu on the right. In this menu, browse for the reconstruction’s destination at the bottom. Please select the correct folder and name the reconstruction as previously done.
18. Go back to the preview tab and select to either start the process or add it to the batch. It is best to press *Add to batch* when you need to do multiple reconstructions.
19. If more reconstructions are needed, repeat the *Starting the reconstruction* steps. When all scans have been selected, press *Start batch*, located on the bottom left, to start all the loaded reconstructions. (These can be found in the list to the right of the *Start batch* button.)
20. If any pop-ups occur after starting the reconstruction or the batch, click okay and continue forward.

The reconstructions are now running.
